# *Paeonia suffruticosa* Andrews root extract ameliorates photoaging via regulating IRS1/PI3K/FOXO pathway

**DOI:** 10.3389/fphar.2025.1520392

**Published:** 2025-03-06

**Authors:** Junxi Liu, Youyun Liu, Feifei Wang, Yonglei Yuan, Hongyu Ma, Liping Qu

**Affiliations:** ^1^ Yunnan Botanee Bio-Technology Group Co., Ltd., Kunming, China; ^2^ Yunnan Characteristic Plant Extraction Laboratory, Yunnan Characteristic Plant Extraction Laboratory Co., Ltd., Kunming, China; ^3^ Botanee Research Institute, Shanghai Jiyan Biomedical Development Co., Ltd., Shanghai, China

**Keywords:** *Paeonia suffruticosa* Andrews root extract (PSAE), polyphenol, paeonol, ultraviolet radiation, anti-photoaging, 3D full T-skin^TM^

## Abstract

**Introduction:**

The root of *Paeonia suffruticosa* Andrews (*P. suffruticosa* Andr.), is a traditional Chinese medicine. Numerous studies have shown that it possesses anti-inflammatory, antioxidant, and anti-aging effects due to its rich content of bioactive compounds such as polyphenols and paeonol. Thus, it finds extensively applied in the fields of medicine and cosmetics. However, there are few reports on the photoprotective effects of *P. suffruticosa* Andr. root bark, this study aims to investigate its research in this area.

**Methods:**

This study utilized *P. suffruticosa* Andr. root bark sourced from Kunming, Yunnan Province, China. The *P. suffruticosa* Andr. root extract (PSAE) was obtained using AB-8 resin. The photoprotective effect of PSAE was assessed using HaCaT cells, HFF cells, and a 3D Reconstructed Human full T-Skin™ model. Mechanistic investigations were performed using RT-qPCR, WB, IF, H&E staining, Masson’s trichrome staining and IHC staining. Finally, an assessment of the effects on humans was conducted.

**Results:**

The total phenolic content in the obtained PSAE was 48.9%. Antioxidant activity studies demonstrated that PSAE effectively inhibits DPPH radicals, superoxide anions, hydroxyl radicals, and ABTS radicals, while also enhancing the inhibition rates of collagenase and hyaluronidase. *In vitro* studies on photoaging resistance revealed that PSAE significantly reduced the UV-induced increases in reactive oxygen species (ROS) levels and senescence-associated β-galactosidase (SA-β-gal) activity. Mechanistic studies indicated that PSAE suppressed the overexpression of IRS1 and its downstream effectors, including PI3K, AKT, and mTOR induced by UV irradiation. A human efficacy assessment was conducted by evaluating parameters such as transepidermal water loss (TEWL), epidermal moisture content, roughness and elasticity, confirming the efficacy of PSAE in humans.

**Discussion:**

In summary, PSAE attenuates UV-induced oxidative damage, genetic damage, and collagen degradation associated with photoaging by modulating the IRS/PI3K/FOXO signaling pathway. This study elucidated the mechanism through which PSAE, thereby providing strong support for its application in cosmetic anti-aging formulations.

## Graphical Abstract

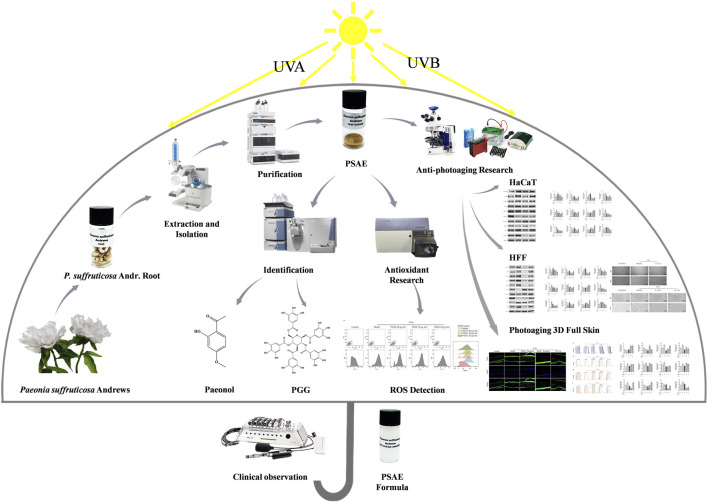

## 1 Introduction

Aging involves a decline in skin function and structure due to both endogenous and exogenous factors. Among these, sunlight-induced ultraviolet radiation is the most significant. Solar UV radiation consists of UVA, UVB, UVC, and UVD. UVD denotes vacuum ultraviolet light, characterized by wavelength range of 100–200 nm. UVC with a wavelength of 200–280 nm are often employed for germicidal applications due to their weak penetration. Both types of UV radiation are largely absorbed by the ozone layer under natural conditions, preventing them from reaching the Earth’s surface and causing biological effects (Wlaschek et al., 2001). The UVB spectrum, ranging from 280 to 320 nm, reaches the skin’s surface and induces sunburn and erythema within a short exposure time. This type of UV radiation can lead to blistering, inflammation, excessive pigmentation, and carcinogenesis. In contrast, the UVA spectrum ranges from 320 to 400 nm, causing skin darkening and penetrating into the dermis and deeper tissues, where it damages elastic fibers, collagen fibers, and other delicate skin structures. This damage manifests as wrinkles, fine lines, and skin laxity, collectively known as photoaging in medical terminology. UVA also significantly impacts pigmentation, reduces skin hyaluronic acid levels leading to dryness, and potentially contributes to the development of skin cancer. Importantly, UVA demonstrates high penetration capabilities, capable of traversing glass, transparent plastics, and similar materials (Svobodova et al., 2006). In summary, UVA and UVB represent the primary exogenous sources of photoaging, capable of inducing temporary or permanent genetic damage within the cytoplasm of skin cells (Gromkowska‐Kępka et al., 2021). This damage directly impacts cellular differentiation, growth, aging, and pathways leading to tissue functional degradation. Ultimately, these effects contribute to cellular senescence and apoptosis. Therefore, this study employed UVA-induced photoaging models in HFF cells and UVB-induced photoaging models in HaCaT cells to comprehensively assess the anti-photoaging potential of PSAE. Ultraviolet radiation exposure on the skin leads to an overproduction of reactive oxygen species (ROS) in cells, which activates the NF-κB pathway through the MAPK, p38, and JNK pathways (Salminen et al., 2022). Upon release, these factors activate the nuclear transcription complex activator protein-1 (AP-1), leading to increased transcription of matrix metalloproteinases (MMPs) and decreased expression of procollagen type I protein and transforming growth factor-β (TGF-β) receptors (Fisher et al., 2002; Salminen et al., 2022). Reduced expression of TGF-β receptors inhibits pro-collagen synthesis, thereby promoting the production of proteolytic enzyme MMP-1. This cyclical process accelerates collagen degradation in the skin, leading to inconsistent dermal matrix structure, decreased skin elasticity, and increased wrinkles, thereby promoting photoaging of the skin. Simultaneously, upon activation, Nrf2 binds with antioxidant response elements (ARE). The Nrf2/ARE pathway activates antioxidant genes like NAD(P)H quinone dehydrogenase 1 (NQO1), heme oxygenase-1 (HO-1), and dihydrolipoamide dehydrogenase (DLD), which help reduce oxidative stress, lower ROS levels, and trigger antioxidant defenses to combat skin photoaging (Wang et al., 2020).

*Paeonia suffruticosa* Andr., a valuable woody plant native to China (Liu et al., 2023). Its medicinal uses have been documented in ancient texts, such as the “Shennong Ben Cao Jing” (The Divine Farmer’s Materia Medica) and “Ben Cao Gang Mu” (Compendium of Materia Medica) dating back over a thousand years. With ongoing research, the active compounds in peony have become increasingly well understood, demonstrating medicinal values including antimicrobial, anti-inflammatory, antioxidant, and immune-regulatory effects (He et al., 2019; Liu et al., 2020; Kim et al., 2023). Given its broad medicinal benefits, there has been growing interest in exploring its potential for skincare and beauty applications. Peony flowers are rich in phenolic compounds and flavonoids, which can resist aging and promote youthful skin (Lin et al., 2019). Peony seed oil is abundant in β-sitosterol and paeoniflorin, which are effective for whitening and brightening skin tone (Wei et al., 2021; Ekiert et al., 2022). Our research team has identified the main active components in the PSAE as PGG (penta-O-galloyl-β-D-glucose) and paeonol (Satoh et al., 1997; Lee et al., 2003; Xu et al., 2006; Sun et al., 2015). Previous studies have found that PGG exhibits strong antioxidant, anti-inflammatory, antiviral, antibacterial, and anti-allergic properties. The potential mechanism of action involves the inhibition of inflammation and oxidative stress via the AMPK/PI3K/Akt/Nrf2 pathway (Wen et al., 2023; Zhang et al., 2022). Paeonol is a phenolic compound with notable antioxidant and anti-inflammatory characteristics (Liu et al., 2021; Li et al., 2023). Studies have reported that paeonol in *P. suffruticosa* Andr. can resist UVB-induced photoaging by modulating the DLD/Nrf2/ARE and MAPK/AP-1 pathways (Sun et al., 2018). However, photoaging caused by UVA should also not be overlooked. Therefore, we have established both UVA and UVB photoaging models to comprehensively explore the anti-photoaging mechanisms of PSAE, providing a theoretical basis for its application as an active ingredient in functional cosmetics.

## 2 Materials and methods

### 2.1 Materials and reagents

The *P*. *suffruticosa* Andr. was procured from Yunnan Yaotong Traditional Chinese Medicine Resources Development Co., Ltd. in Kunming, Yunnan province, China. Researcher Haiyang Liu from the Kunming Institute of Botany identified it as the root bark of *P. suffruticosa* Andr. (JY20221115), belonging to the family Paeoniaceae. The specimen was assigned the identifier BTN-RM-20221115 and deposited in the herbarium of the Botany Research Institute at Shanghai Jiyan Biomedical Development Co., Ltd. Ethanol and butylene glycol were obtained from Sinopharm Chemical Reagent Co., Ltd., while acetonitrile, collagenase (from porcine pancreas), N-methoxysuccinyl-Ala-Ala-Pro-Val for nitrobenzene, celecoxib, DMSO, PBS, hyaluronidase, sodium hyaluronate, calcium chloride, sodium hydroxide, acetate buffer, p-(dimethylamino) benzaldehyde, potassium tetraborate tetrahydrate, and other reagents were sourced from Sigma-Aldrich. Ultrapure water was obtained from Milli-Q ix7005 system by Merck Millipore. Danshensu, PGG reference standard, ferulic acid, and analytical grade sodium carbonate were sourced from Shanghai Yuan Ye Biotechnology Co., Ltd. AB-8 macroporous resin was obtained from Cangzhou Maoquan New Material Technology Co., Ltd. DPPH (1,1-Diphenyl-2-picrylhydrazyl) and gallic acid (99.1% purity, batch K1819078) were acquired from Shanghai Aladdin Bio-Chem Technology Co., Ltd. ABTS (2,2′-Azino-bis (3-ethylbenzothiazoline-6-sulfonic acid)) was purchased from Nanjing Jiancheng Bioengineering Institute. The CCK-8 kit was obtained from Dojindo Laboratories (Japan), and the RT-qPCR primers were designed and synthesis by Generay Biotech Co., Ltd. (Shanghai, China). RNA was purified using the GeneJET RNA Purification kit (Thermo Fisher Scientific, United States). For RNA reverse transcription, we employed the PrimeScript™ RT kit (Takara, Japan). ELISA kits were obtained from Elabscience Biotechnology Co., Ltd. (Wuhan, China) and all chemicals used in this study were of analytical grade or higher. Masson Dyeing Solution Set, 20× antigen repair solution (pH 6.0, 8.0, 9.0), Hematoxylin staining Solution and DAB chromogener were obtained from Servicebio (Wuhan, China).

### 2.2 Preparation and chemical composition analysis of PSAE

The dried *P*. *suffruticosa* Andr. root bark (200 g) was immersed in a 2 L ethanol-water solution (v/v, 85%) and subjected to reflux extraction in three cycles, each lasting 1.5 h, 1 h, and 1 h respectively. The extracts were combined, concentrated under reduced pressure, and set aside. AB-8 resin was utilized for column chromatography, where the concentrated extract was loaded onto the column. Sequential elution was performed using water, ethanol-water solutions (v/v, 10%, 20%, 80%). The fraction eluted with ethanol-water (v/v, 80%) was collected, concentrated, and dried to obtain the PSAE. The PSAE powder was precisely measured and dissolved in 30% propylene glycol to create a solution with a concentration of about 4 mg ·mL^−1^. The paeonol reference standard was precisely weighed and dissolved in anhydrous ethanol to create a solution with a concentration of approximately 0.75 mg ·mL^−1^. The PGG reference standard was precisely weighed and dissolved in 30% propylene glycol to create a solution with a concentration of approximately 0.75 mg ·mL^−1^. Analysis was conducted using an Agilent 1290U HPLC system with a Poroshell SB-C18 column (2.7 μm, 3 × 100 mm). The temperature was set to 35°C, with detection performed at 274 nm. The flow rate was established at 0.4 mL min^−1^. The chromatographic conditions were set as follows: from 0 to 3 min, 90% solvent A (0.1% formic acid) and 10% solvent B (acetonitrile); 23 min, 75% A and 25% B; 29 min, 63% A and 37% B; from 30 to 34 min, 5% A and 95% B; and from 35 to 40 min, 90% A and 10% B. The peak areas of each injection were accurately recorded, and a standard curve was generated using Excel.

Gallotannic acid was precisely weighed and dissolved in deionized water to create solutions with concentrations of 250.0, 125.0, 62.5, 31.3, and 15.6 mg L^−1^. PSAE was dissolved in deionized water to achieve a concentration of 250.0 mg L^−1^. A 96-well plate was used to test each concentration of the standard solutions and the PSAE solution, with 100 μL of each added to separate wells in triplicate. 100 μL of 1 M Folin-Ciocalteu reagent was added to each well, mixed thoroughly, and reacted at room temperature for 5 min. Subsequently, 100 μL of 15% sodium carbonate solution was added, mixed thoroughly, and incubated at room temperature in the dark for 1 h. Absorbance at 760 nm was measured for each well. Deionized water served as a blank control to establish the standard curve and regression equation for gallotannic acid, which was then used to calculate the absorbance of the PSAE.

### 2.3 Antioxidant assays in vitro

#### 2.3.1 DPPH assay

The DPPH assay was employed to evaluate the scavenging activity of PSAE, following a modified procedure based on Luis Cartuche (Cartuche et al., 2024). Precisely weigh 10.5 mg of 1,1-diphenyl-2-picrylhydrazyl (DPPH), dissolve it in anhydrous ethanol, and make up the volume to 100 mL. For PSAE, weigh 50.00 mg, dissolve it in 30% butylene glycol to a final volume of 50 mL to prepare a 1.00 mg/mL PSAE solution. This solution is then diluted with 30% butylene glycol to obtain various concentration gradients (0–1.00 mg/mL). Add 2 mL of each concentration of PSAE solution to separate test tubes, followed by the addition of 2 mL of DPPH solution. After thorough mixing, incubate the mixtures at room temperature for 30 min. Measure the absorbance at 517 nm and calculate the scavenging activity using Formula 1. The blank control consisted of 4 mL of 40% ethanol solution, with absorbance denoted as A0; the sample group absorbance is denoted as A1; and the control group absorbance is denoted as A2. Vitamin C was used as a reference, and measurements were performed using the same procedure.

$$\text{DPPH Scavenging activity }\%=\frac{A_{2}-A_{1}}{A_{2}-A_{0}}\times 100\%$$ (1)

#### 2.3.2 ABTs assay

The ABTS assay, adapted from Luis Cartuche’s method (Cartuche et al., 2024), was employed to assess the scavenging activity of PSAE. Precisely weigh 50.00 mg of the PSAE, dissolve it in 30% butylene glycol to a final volume of 50 mL to obtain a 1.00 mg/mL extract solution, and further dilute with 30% butylene glycol to prepare solutions of varying concentrations (0–1.00 mg/mL). For the control, weigh 10.2 mg of vitamin C, dissolve it in distilled water to make a 0.51 mg/mL solution, and then dilute with distilled water to prepare solutions of different concentrations (0–0.51 mg/mL). The prepared samples and controls were tested for ABTS scavenging activity according to the kit’s instructions. Scavenging activity was determined using Formula 2, with A1 as the absorbance of the reaction mixture post-sample addition and A0 as the absorbance without the sample.

$$\text{ABT}s\text{ Scavenging activity }\%=\frac{A_{0}-A_{1}}{A_{0}}\times 100\%$$ (2)

#### 2.3.3 Collagenase inhibition assay

The collagenase inhibition assay, adapted from Hering A (Hering et al., 2023), was employed to assess the inhibitory activity of PSAE. The substrate N-methoxy-succinyl-Ala-Ala-Pro-Val p-nitroanilide was utilized, with 1X PBS buffer (pH 7.2–7.4) containing sodium sivelestat serving as the positive control. The reaction mixture comprised PBS buffer, 15.625 μg/mL porcine pancreatic collagenase, and PSAE at concentrations ranging from 0 to 1 mg/mL, dissolved in 10% DMSO. The control reaction mixture comprised PBS buffer and 15.625 μg/mL porcine pancreatic elastase. The reaction mixture was pre-incubated at 37°C for 10 min before initiating the reaction by adding 0.1 mM substrate, followed by a 15-min incubation at the same temperature. A Multiskan SkyHigh microplate reader (BIOCOM SYSTEMS) was used to detect changes in 4-nitroaniline on a 96-well plate at 405 nm. The results were derived using Formula 3, with A as the OD value of the control solution, B as the OD value of the control blank, C as the OD value of the sample solution, and D as the OD value of the sample blank.

$$\text{Collagenase Inhibition ratio }\%=\frac{\left(A-B\right)-\left(C-D\right)}{A-B}\times 100\%$$ (3)

#### 2.3.4 Hyaluronidase assay

The Hyaluronidase assay, modified from Hering A’s method, was employed to assess the inhibitory activity of PSAE (Hering et al., 2023). Hyaluronidase from bovine testicles was employed with hyaluronic acid as the substrate, and glycyrrhizic acid dipotassium salt was used as the positive control. Acetate buffer (0.1 mM, pH 7.0) was prepared using acetic acid and sodium acetate. The sample solutions contained varying concentrations of the sample (0–100 μg/mL) and hyaluronidase (0.3 mg/mL) dissolved in acetate buffer (0.1 mM, pH 7.0). The control solution included 0.3 mg/mL of hyaluronidase. The mixtures were incubated at 37°C for 20 min, then calcium chloride (12.5 mM) was added, and the incubation continued at 37°C for another 20 min to activate the hyaluronidase. The reaction was initiated by adding sodium hyaluronate (1 mg/mL, in acetate buffer) and incubated at 37°C for 40 min. The reaction was halted by adding 0.4 M sodium hydroxide and 0.4 M potassium borate, then heated at 85°C for 5 min, cooled on ice for 2 min, and left at room temperature for 5 min. The reaction mixture was transferred to a 96-well plate (BIOCOM SYSTEMS), and 2-dimethylamino benzaldehyde solution (32 mg/mL) was added in a 1:1 ratio with the reaction mixture. The absorbance (OD value) was measured at 585 nm after shaking at 37°C for 1 min and allowing the sample to stand at room temperature for 10 min. Under alkaline conditions, β-N-acetylglucosamine, generated from hyaluronic acid by hyaluronidase hydrolysis, reacts with dimethylamino benzaldehyde to form a purple-colored derivative, 2-methyl-3-diacetylpyrrole. The control solution contained only hyaluronidase and hyaluronic acid without the sample. Blank controls consisted of acetate buffer (0.1 mM, pH 7.0), calcium chloride (12.5 mM), sodium hydroxide (0.4 M), and potassium borate (0.4 M). The hyaluronidase inhibition rate (%) was determined using Formula 4, with A representing the OD value of the sample solution, B the OD value of the sample blank, C the OD value of the control solution, and D the OD value of the control blank.

$$\text{Hyaluronidase Inhibition rate }\%=\frac{\left(C-D\right)-\left(A-B\right)}{C-D}\times 100\%$$ (4)

### 2.4 Antiphotoaging assays in vitro

#### 2.4.1 Cell culture

HaCaT and HFF cells, sourced from the Chinese Academy of Sciences Cell Bank, were cultured in DMEM supplemented with 10% heat-inactivated FBS. Cells were cultured in a humidified incubator at 37°C with 5% CO_2_. The medium was refreshed every 2–3 days. When the cells in a 100 mm dish reached 80%–90% confluency, they were digested with trypsin and subsequently subcultured at a 1:3 ratio. Cell viability was evaluated using the CCK-8 assay kit according to the manufacturer’s instructions.

#### 2.4.2 UV-induced cellular photoaging model

Cells in good growth condition and in the exponential growth phase were placed in a 3T3 NRU phototoxic UV irradiator. In the UVB model, HaCaT cells underwent UVB exposure at 0.2 mW/cm^2^ intensity and 12 mJ/cm^2^ energy dose. In the UVA model, HFF cells were exposed to UVA irradiation with an intensity of 2 mW/cm^2^ and an energy dose of 15 J/cm^2^. The irradiation time was calculated using the Formula 5. After irradiation, fresh complete medium was added.

$$\text{UV energy }\left({\text{mJ}/\text{cm}}^{2}\right)=\text{irradiation time }\left(s\right)\times \text{UV intensity }\left(\text{mW}/{\text{cm}}^{2}\right)$$ (5)

#### 2.4.3 UVA-induced 3D Reconstructed Human full T-Skin™ photoaging model

This study employed T-Skin^®^ from Sianfunuo Biotechnology Co., LTD. (Shanghai, China), a commercially available *in vitro* human epidermis model. T-Skin^®^ is cultured on collagen at the air-liquid interface, closely mimicking the human epidermis as shown by histological analysis. After pre-incubation, the tissues were treated with PBS for 10 min, followed by washing with DPBS. The tissues were then exposed to UVA irradiation to create a UVA-induced 3D skin photoaging model. After irradiation, the medium was replaced. The tissues were incubated with different concentrations of PSAE mixture, using a volume of 20 μL. After 24–48 h of treatment, the tissues were washed again with DPBS and reserved. The main components of the basic cosmetic formula include water, acrylates, dimethicone, tridecyl trimellitate, glycerin, PEG-100 glyceryl stearate, xanthan gum, pentylene glycol, and aminomethyl propanol.

#### 2.4.4 ROS detection

The experiment utilized cells in optimal growth conditions during the exponential phase, categorized into a blank group, model group, negative control group, and PSAE intervention group. The blank, model, and negative control groups were incubated with cell culture medium, while the PSAE intervention group was incubated with complete medium containing the appropriate concentration of the test substance for 2 h in an incubator. PBS was then substituted for the medium, and UV irradiation was performed using a 3T3 NRU phototoxic UV irradiator, except for the blank and negative control groups. After irradiation, the PSAE group was further incubated with PSAE for 1 h. ROS levels in each group were measured using flow cytometry, following the instructions provided with the ROS assay kit.

#### 2.4.5 SA-β-gal detection

To evaluate the effects of photodamage, we utilized the SA-β-gal Staining Kit (C0602) from Beyotime Biotechnology Co., Ltd. (Shanghai, China). The procedure was followed the manufacturer’s guidelines. Following overnight incubation at 37°C with a pH 6 staining solution, the study was categorized into a blank control group, a photodamage model group, and a PSAE treatment group. Cells were observed under an inverted microscope at magnifications of 100× and 40×. After capturing images, the percentage of blue-stained cells in each group was determined using ImageJ software.

#### 2.4.6 RT-qPCR analysis

RNA was extracted from cells or 3D skin using an RNA extraction kit. The quality and concentration of RNA samples were measured using a NanoDrop spectrophotometer. The reverse transcription process was performed using the PrimeScript™ RT kit (Takara, Japan). The primer sequences used in the experiment are detailed in Supplementary Table S1. Roche DNA Green Master (Swit) fluorescent dye was added, and detection was carried out using the Roche LightCycler 96 (Swit) real-time PCR amplification conducted. All gene expression data were collected during the linear amplification phase. PCR efficiencies ranged from 90% to 110%. Each sample was analyzed in at least three biological replicates. The mRNA levels of the target genes were quantified using the 2^−ΔΔCT^ method, with β-actin serving as the normalization reference.

#### 2.4.7 Western blot analysis

After incubating the cells for 24 h, they were washed twice with pre-chilled PBS. All consumables and reagents were pre-chilled, and procedures were conducted on ice. Unless stated otherwise, reagents were sourced from Beyotime Biotechnology Co., Ltd., Shanghai, China. Cells were lysed on ice with a lysis buffer supplemented with protease and phosphatase inhibitors to produce cell lysate. The lysate underwent centrifugation at 14,000 g for 10 min at 4°C. Protein levels were measured with a BCA Protein Assay Kit, and equivalent protein quantities were heated in SDS-PAGE sample buffer at 95°C for 5 min. The protein samples were then separated using SDS-PAGE with pre-cast gels. After separation, proteins were transferred to PVDF membranes (Millipore, Burlington, MA, United States), followed by washing and blocking. Membranes were initially treated with specific primary antibodies (β-actin, PI3K/P-PI3K AB191606/AB278545, AKT/P-AKT AB179463/AB192623, mTOR AB134903, FOXO/P-FOXO AB109629/AB154786, Nrf2 AB62352, IRS/P-IRS AB40777/AB178703), and subsequently with HRP-conjugated secondary antibodies. Immunodetection utilized Chemistar™ High-sig ECL Western blotting Substrate (Tanon Life Science, Shanghai, China). Images were obtained using the Tanon-5200 Chemiluminescent Imaging System (Tanon Life Science, Shanghai, China) and analyzed with ImageJ software to assess relative immunoreactivity.

#### 2.4.8 H&E stained and immunofluorescence analysis

The 3D Reconstructed Human full T-Skin™ tissue was carefully removed from the Transwell insert using a sterile surgical blade, separated, and cut into two-halves. Samples were fixed overnight in 4% paraformaldehyde. The samples underwent dehydration using ethanol and xylene before being embedded in paraffin. The tissue was cut into 5 μm slices for staining and immunofluorescence analysis.

The slices underwent rehydration following paraffin removal and were subsequently stained with hematoxylin and eosin for H&E staining. Images were obtained with a ZESS Axio Scope microscope from Germany. Epidermal thickness (ET) was measured using the ZESS application suite, and the images were analyzed and quantified with ImageJ software.

For immunofluorescence detection, paraffin was removed from the sections, which were then rehydrated through a series of decreasing ethanol concentrations. The slices were subjected to a 20-min treatment with heated citrate buffer (pH 6.4) followed by cooling to room temperature. The sections underwent three 5-min washes with phosphate-buffered saline (PBS, pH 7.4) to permeabilize the tissue. Blocking was conducted for 30 min using a PBS solution with 1.5% bovine serum albumin (BSA) and 0.025% TritonX-100. The primary antibodies (Abcam, Cambridge, United Kingdom: anti-IRS1, anti-PI3K, anti-FOXO, anti-COL3, anti-COL4, anti-NRF2, anti-SOD. Anti-FOXO and anti-GADD45 antibodies from Sigma-Aldrich, Missouri, United Kingdom, were incorporated into the blocking solution. The sections were incubated overnight at 4°C with the primary antibody, then washed three times with PBS (pH 7.4) for 5 min each. The sections were then incubated with a 1:2500 dilution of secondary antibody for 1 h at room temperature. After three washes with PBS for 5 min each, the slices were treated with DAPI and then washed with PBS three additional times, each for 5 min. The sections were mounted with a fluorescence mounting medium. Immunofluorescence images were obtained with a ZESS Axio Scope microscope.

#### 2.4.9 Masson’s trichrome stained and immunohistochemistry analysis

For Masson’s trichrome staining, the 3D Reconstructed Human full T-Skin™ tissue were fixed in 4% paraformaldehyde, embedded in paraffin, and subsequently deparaffinized through a series of graded alcohols to water. The sections were stained with Masson’s trichrome stain, differentiated using 1% acetic acid, and dehydrated with absolute ethanol. Following this, the sections were cleared in xylene and mounted with neutral gum. Microscopic examination and image acquisition were performed using a Nikon Eclipse E100 microscope equipped with a Nikon DS-U3 imaging system.

For immunohistochemical staining, the 3D Reconstructed Human full T-Skin™ tissue underwent identical fixation and embedding procedures as described above. Antigen retrieval was conducted by immersing the slides in an EDTA buffer (pH 8.0) at 90°C for 30 min, followed by natural cooling. Slides were then washed three times in PBS (pH 7.4) on a decolorization shaker for 5 min each. Endogenous peroxidase activity was blocked by incubating the slides in 3% hydrogen peroxide solution at room temperature in the dark for 25 min, followed by another set of three PBS washes. Sections were blocked with 3% BSA for 30 min at room temperature. Primary antibodies against COLI (Servicebio GB154197), COLIII (Servicebio GB111629), and COLIV (Servicebio GB115688) were applied and incubated overnight at 4°C in a humidified chamber. After washing the sections three times in PBS, secondary antibodies specific to the primary antibodies (HRP-labeled goat anti-rabbit IgG (Servicebio GB23303) and HRP-labeled goat anti-mouse IgG (Servicebio GB23301)) were added and incubated at room temperature for 50 min. The slides were incubated in PBS (pH 7.4) and washed three times on a decolorizing shaker for 5 min each. Following this, the slides were air-dried briefly before being treated with freshly prepared DAB chromogen solution. Color development was monitored under a microscope and terminated by rinsing the sections with deionized water. Finally, hematoxylin staining was performed. After dehydration and clearing, the slides were allowed to air-dry slightly, sealed with mounting medium, examined under a microscope (NIKON ECLIPSE E100, Japan), and images were captured and analyzed using an imaging system (NIKON DS-U3, Japan).

#### 2.4.10 ELISA assay

COL1 and COL3 expression levels were measured using an ELISA kit from Elabscience Biotechnology Co. Ltd., Wuhan, Hubei, China. Supernatants from each cell culture group were collected, centrifuged at 1,000 g for 5 min to eliminate cell debris, and subsequently transferred to new tubes for storage. Experiments were conducted in triplicate following the manufacturer’s guidelines.

### 2.5 Human efficacy evaluation

The Ji Yan Ethics Committee reviewed and approved this study (JYE20231201), consenting to its conduct in accordance with the approved trial protocol, informed consent form, recruitment materials, and related documents. The study complied with the Declaration of Helsinki, Good Clinical Practice guidelines, and was rigorously conducted in line with the research protocol and relevant operational procedures.

Eligible participants were aged 30 to 50, understood the trial, voluntarily consented, signed informed consent, and adhered to testing protocols.

Participants were placed in a controlled setting with a temperature range of 20°C–22°C and humidity levels between 40% and 60% RH for 30 min. Testing was conducted on the forearms of both hands. A total of six test areas (3 cm × 3 cm) were marked on the forearms, three on each side, ensuring statistical balance of all sample locations. Samples were applied once at a dosage of (2.0 ± 0.1) mg/cm^2^, using latex finger cots to evenly spread the sample within the test area, and randomly distributed across the designated areas on both arms.

Antera 3D imaging, transepidermal water loss (TEWL), epidermal moisture content, roughness, and elasticity were assessed at baseline and 2, 4, and 6 h post-application. The cosmetic base formula used is consistent with that employed in Section 2.4.3 of the study.

### 2.6 Statistical analysis

In this study, results are presented as mean ± SEM. Data analysis utilized GraphPad Prism 7 and SPSS 26.0. In human efficacy testing, paired sample t-tests were employed for within-group comparisons when data followed a normal distribution; otherwise, rank-sum tests were utilized. All statistical analyses were conducted as two-tailed tests.

The Shapiro-Wilk test assessed normality, One-way ANOVA tested significance, Levene’s test checked variance homogeneity, and post hoc analyses were conducted using the Tam Heini and LSD tests. The significance level was established at α = 0.05, where *p* ≤ 0.05 (*) denotes significance, *p* < 0.01 (**) indicates greater significance, and *p* < 0.001 (***) represents the highest significance.

## 3 Results

### 3.1 Analysis of chemical contents of PSAE

**FIGURE 1:**
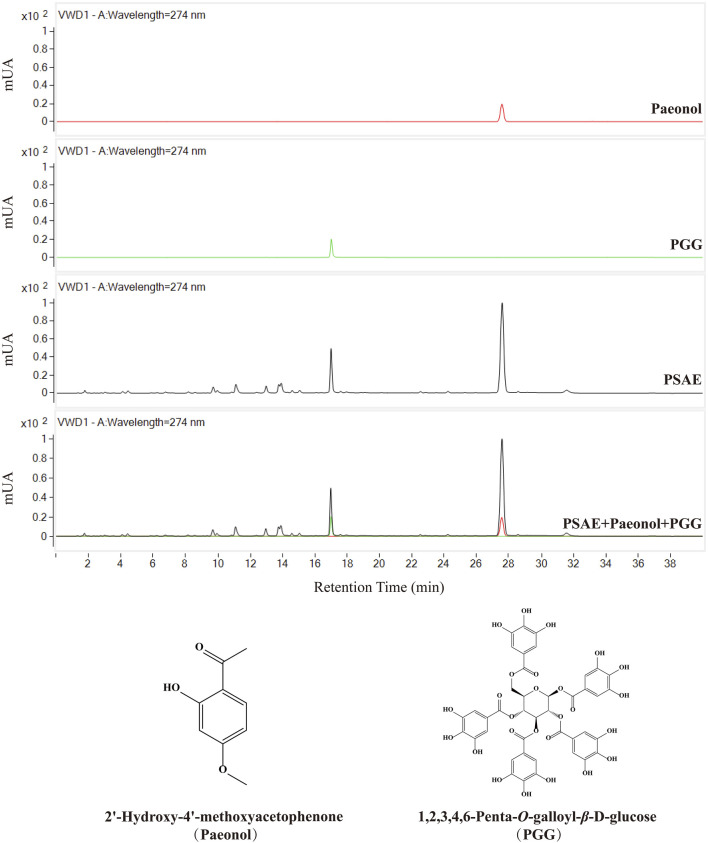
HPLC chromatograms of the PSAE and two reference standards, Chemical structures of Paeonol and PGG.

This study employed HPLC to analyze the PSAE, with the results presented in Figure 1. The high-resolution mass spectrometer was also used for further qualitative analysis of the active substances, with detailed results referenced in Supplementary Figure S1. After comparing the chromatographic peaks, the characteristic results of the standard reference compounds were consistent with those in PSAE. Based on this, it was concluded that the main components of PSAE obtained under the conditions of this study are PGG and paeonol.

The total phenolic content in the PSAE was 48.9%. A standard curve was constructed based on the relationship between peak area and injection volume for two reference standards. The standard curve for PGG is represented by the equation *y* = 5,296.6× −44.369, and for paeonol, the equation is *y* = 8,480.7×−5.7871. The calculated contents of PGG and paeonol in the JY211115 batch of the PSAE were 10.79% and 23.85%, respectively.

### 3.2 Antioxidative evaluation result *in vitro*

**FIGURE 2:**
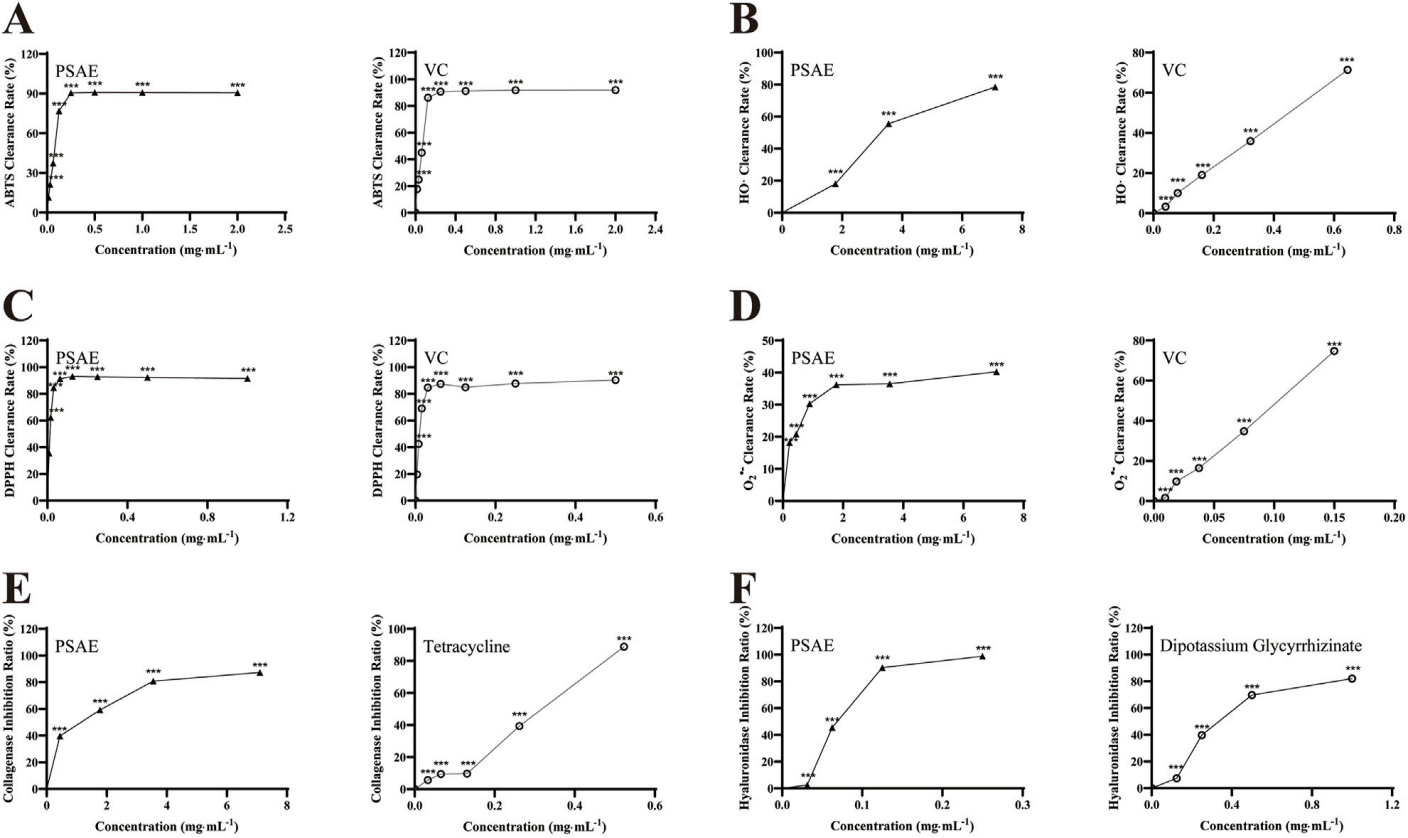
Study on the antioxidant efficacy of PSAE. **(A)** ABTS radical scavenging activity **(B)** Hydroxyl radical scavenging activity **(C)** DPPH radical scavenging activity **(D)** Superoxide anion scavenging activity **(E)** Collagenase inhibition rate **(F)** Hyaluronidase inhibition rate. n = 3. Compared to the control group, *p < 0.05, **p < 0.01, ***p < 0.001.

This study evaluated the antioxidant activity of PSAE by analyzing six biochemical indicators: DPPH, superoxide anion, hydroxyl radical, and ABTS radical scavenging activities, along with collagenase and hyaluronidase inhibition rates. The experimental results are shown in Figure 2. PSAE was compared with Vitamin C (VC), Tetracycline, and Dipotassium Glycyrrhizinate, and the results indicated that its efficacy is similar to that of the positive control group. It efficiently suppresses DPPH, superoxide anions, hydroxyl, and ABTS radicals, and enhances the inhibition rates of collagenase and hyaluronidase. Therefore, preliminary analysis suggests that PSAE possesses antioxidant properties.

### 3.3 Antiphotoaging effect of PSAE on UVB-exposed HaCaT cells

**FIGURE 3:**
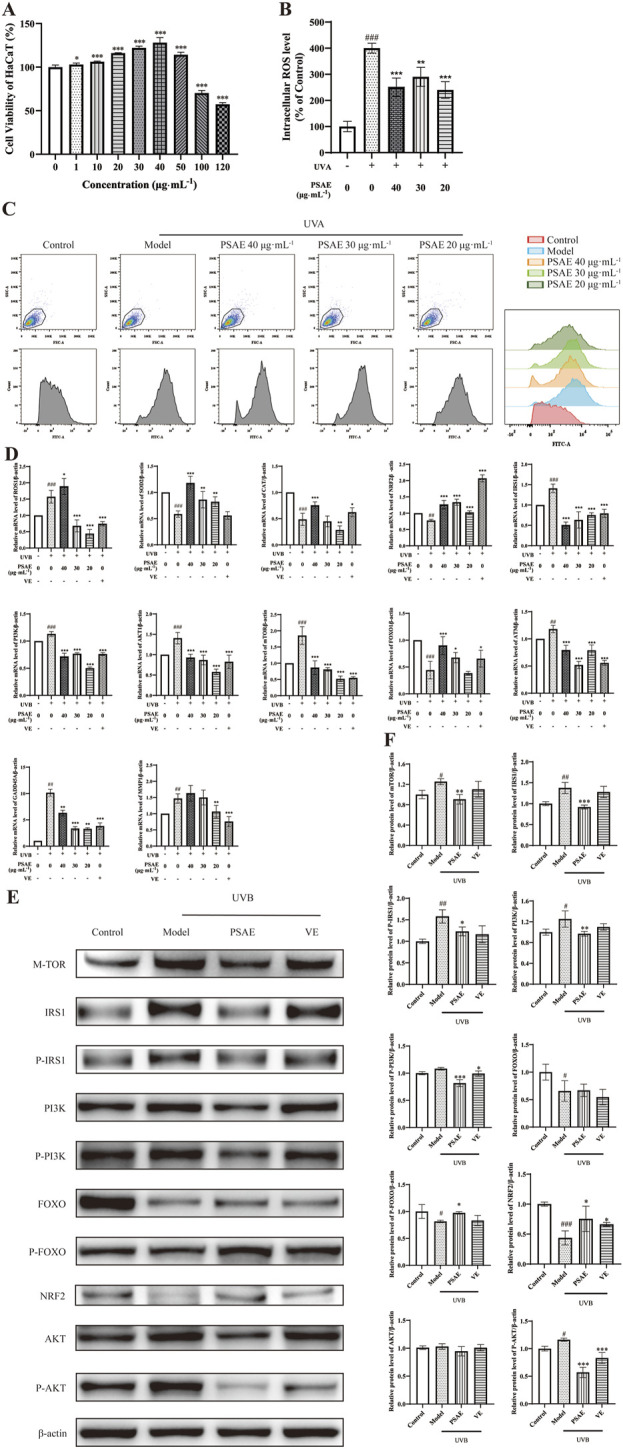
Study of the anti-photoaging effects of PSAE on UVB-irradiated HaCaT cell photoaging model. **(A)** HaCaT cells were treated with PSAE (0–120 μg/mL) for 24 h, and cell viability was assessed using the CCK-8 assay. **(B)** Semi-quantitative histogram of PSAE’s effect on cellular ROS levels detected by flow cytometry, n = 3. **(C)** Peak graph, Scatter plot and FITC fluorescence signal peak plot of PSAE’s effect on cellular ROS levels detected by flow cytometry. **(D)** RT-qPCR analysis of PSAE’s effect on the mRNA levels of ROS1, SOD2, CAT, Nrf2, IRS1, PI3K, AKT1, mTOR, FOXO, ATM, GADD45A, and MMP1. n = 4. **(E)** WB analysis of PSAE’s effect on protein levels of mTOR, P-/IRS1, P-/PI3K, P-/FOXO, Nrf2, and P-/AKT. **(F)** Semi-quantitative histogram of protein levels detected by WB, n = 3. Data are expressed as mean ± SEM. Compared to the control group, #*p* < 0.05, ##*p* < 0.01, ###*p* < 0.001; compared to the UVB-irradiated model group, *p < 0.05, **p < 0.01, ***p < 0.001.

As shown in Figure 3A, cell viability of HaCaT cells treated with PSAE was assessed using the CCK-8 assay. PSAE treatment for 24 h at concentrations under 50 μg/mL showed no significant toxicity. At concentrations above 100 μg/mL, PSAE significantly reduced the viability of HaCaT keratinocytes (Gao et al., 2020).

ROS detection results are shown in Figures 3B, C. Pre-treating HaCaT cells with PSAE at concentrations of 20–40 μg/mL resulted in a decrease in intracellular ROS levels compared to the oxidative stress model group. This indicates that PSAE can reduce the surge in ROS generation caused by UVB radiation.

RT-qPCR analysis results are presented in Figure 3D. In the UVB-irradiated HaCaT photoaging model, PSAE demonstrated antioxidant properties by significantly reducing ROS1 expression and enhancing the expression of antioxidants SOD2, CAT, and the oxidative stress-related factor Nrf2, compared to the model group. PSAE modulated gene expression by decreasing IRS1, AKT1, PI3K, and mTOR levels, while increasing FOXO expression, relative to the model group. This suggests that PSAE promotes FOXO expression and inhibits mTOR expression by suppressing the IRS1/AKT1/PI3K pathway. PSAE reduced ATM and GADD45A expression relative to the model group, suggesting its role in inhibiting ATM to lower GADD45A levels, thus providing an anti-DNA damage effect. PSAE reduced MMP1 expression relative to the model group.

Western blot detection results are shown in Figures 3E, F. The PSAE treatment group notably decreased the protein expression levels of mTOR, P-IRS1/IRS1, PI3K, and P-AKT, while significantly increasing the expression of P-FOXO and Nrf2 compared to the model group. These findings align with the regulatory influence of PSAE on gene expression.

### 3.4 Antiphotoaging effect of PSAE on UVA-exposed HFF cells

**FIGURE 4:**
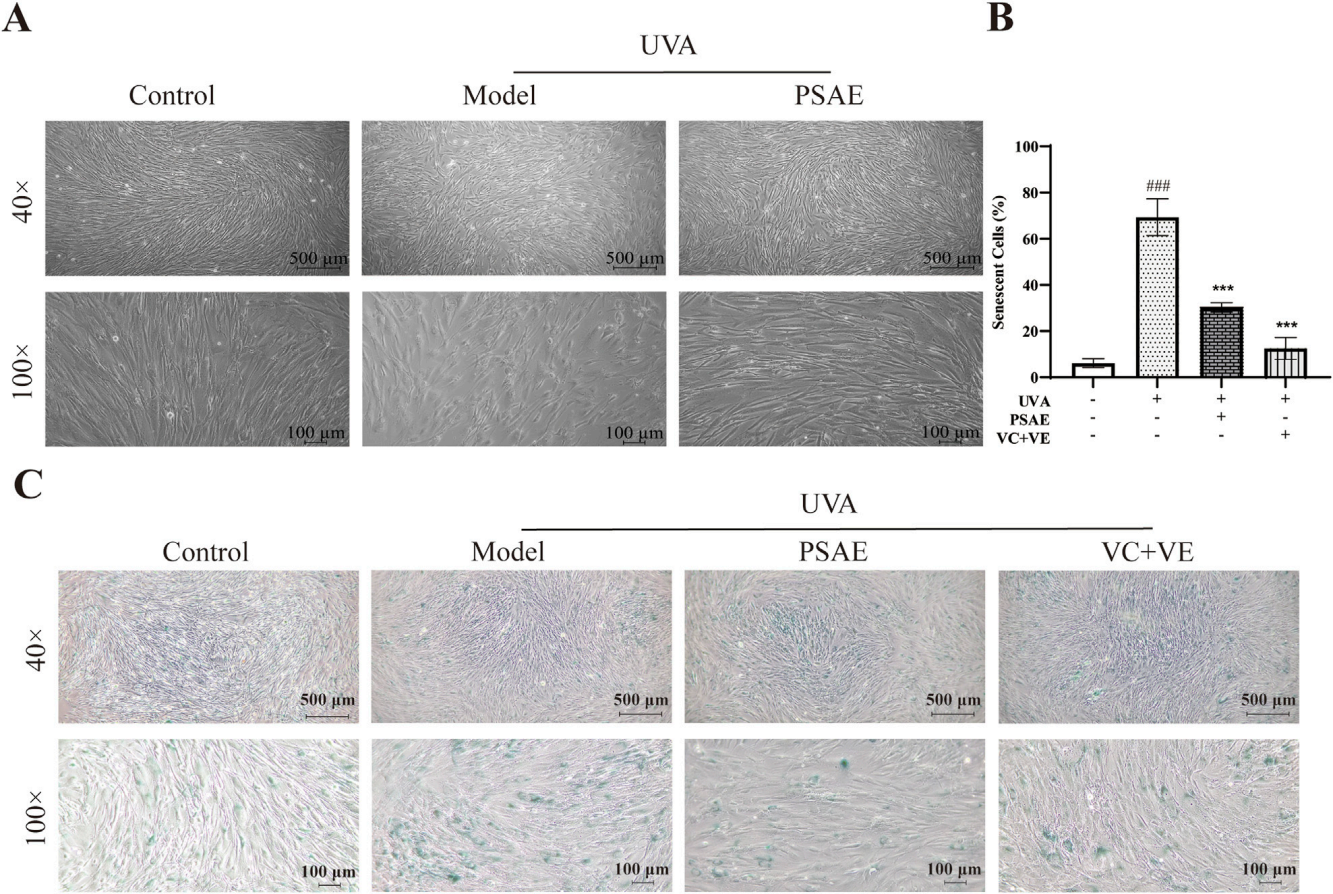
β-gal staining analysis was conducted to study the photoaging model of HFF cells induced by UVA irradiation (40×, 100×). **(A)** Morphological changes of HFF cells induced by UVA under a microscope **(B)** Semi-quantitative calculation results of β-gal staining, n = 3 **(C)** β-gal staining detection results under a microscope. Statistical significance was determined as follows: compared to the blank control group, ###*p* < 0.001; compared to the UVA irradiation model group, ***p < 0.001.

To demonstrate the anti-photoaging effects of PSAE at a macroscopic level, including overall cellular morphology and SA-β-gal, a well-established indicator of senescence, the following experiments were conducted. The results of the microscopy and SA-β-gal detection experiments are shown in Figure 4. The Model group showed reduced cell numbers, increased nuclear size, and morphological changes from swollen fusiform to elongated round shapes compared to the Control group. The PSAE group mitigated these morphological changes, indicating that PSAE has protective effects against light-induced damage in Figure 4A (Yamaba et al., 2016). β-gal staining was performed on HFF cells before and after photoaging in Figures 4B, C. Compared to the blank group, the staining intensity was significantly enhanced in the model group, indicating β-galactosidase production in aging cells. PSAE intervention significantly reduced aging cells and staining intensity compared to the model group, indicating a protective effect against UVA-induced photoaging in HFF cells, as quantified by ImageJ.

**FIGURE 5:**
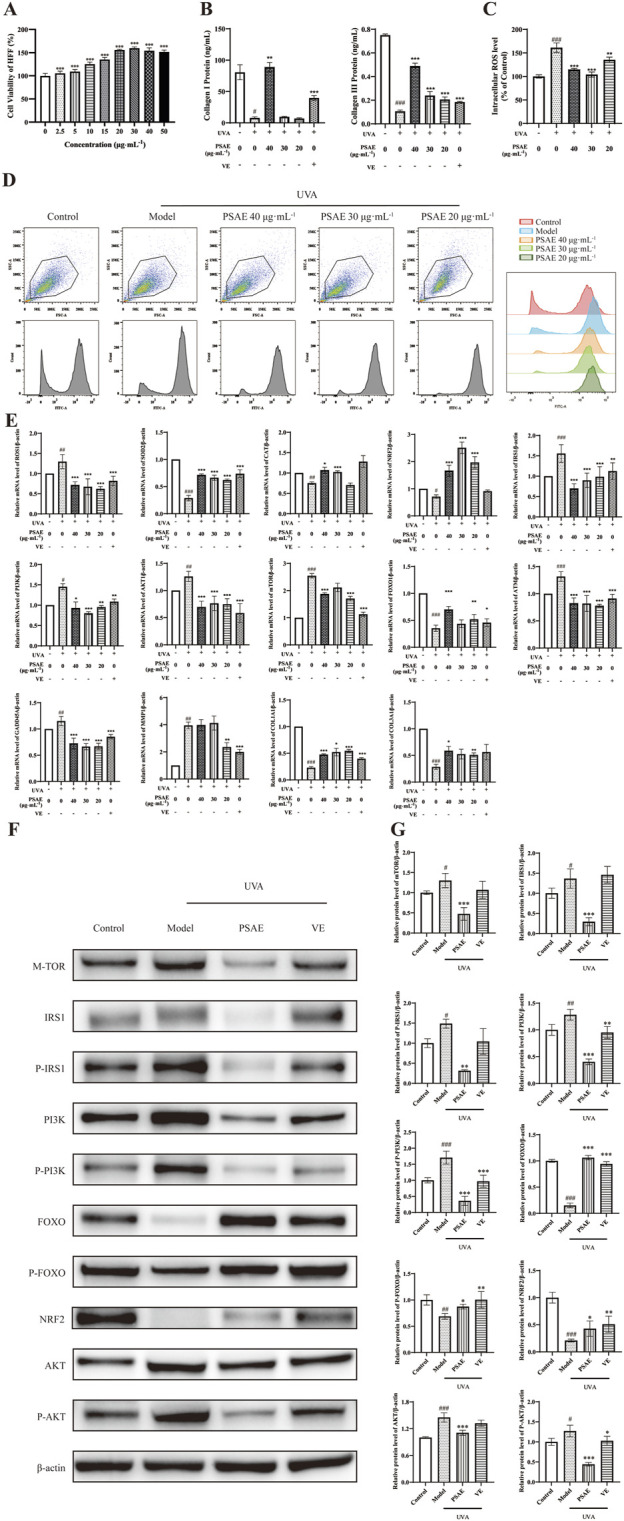
Study of the anti-photoaging effects of PSAE on the UVA irradiation-induced HFF cell photoaging model. **(A)** Effect of PSAE on HFF cell viability. Cells were treated with PSAE (0–50 μg/mL) for 24 h, and cell viability was assessed using the CCK-8 assay **(B)** ELISA was used to measure the effects of PSAE on the expression levels of COL1 and COL3 proteins (n = 3) **(C)** Semi-quantitative bar graph showing the impact of PSAE on cellular ROS levels as detected by flow cytometry (n = 3) **(D)** Peak graph, Scatter plot and FITC fluorescence signal peak graph showing the impact of PSAE on cellular ROS levels as detected by flow cytometry **(E)** RT-qPCR analysis of the effects of PSAE on mRNA levels of ROS1, SOD2, CAT, Nrf2, IRS1, PI3K, AKT1, mTOR, FOXO, ATM, GADD45A, MMP1, COL1A1, and COL3A1 (n = 4) **(F)** WB analysis of the effects of PSAE on protein levels of mTOR, P-/IRS1, P-/PI3K, P-/FOXO, Nrf2 **(G)** Semi-quantitative bar graph showing the impact of PSAE on protein levels as detected by WB (n = 3). Data are presented as mean ± SEM. Statistical significance was determined as follows: compared to the blank control group, #*p* < 0.05, ##*p* < 0.01, ###*p* < 0.001; compared to the UVA irradiation model group, *p < 0.05, **p < 0.01, ***p < 0.001.

Cell viability of HFF cells treated with PSAE was assessed using the CCK-8 method. The findings presented in Figure 5A shows that PSAE treatment for 24 h does not exhibit significant toxicity at concentrations under 15 μg/mL. At concentrations above 20 μg/mL, PSAE significantly reduced the viability of HFF cells (Gao et al., 2020).

ROS results are shown in Figures 5C, D. Pre-treating HFF cells with PSAE at concentrations of 20–40 μg/mL reduced intracellular ROS levels relative to the oxidative stress model group. This suggests that PSAE can reduce the surge in ROS generation induced by UVA radiation.

RT-qPCR analysis results are shown in Figure 5E. In the UVA-irradiated HFF photoaging model, PSAE demonstrated antioxidant effects by significantly decreasing ROS1 expression and increasing the expression of antioxidants SOD2, CAT, and the oxidative stress-related factor Nrf2 compared to the Model group. PSAE modulated gene expression by decreasing IRS1, AKT1, PI3K, and mTOR levels and increasing FOXO expression compared to the Model group. This suggests that PSAE promotes FOXO expression and inhibits mTOR expression by suppressing the IRS1/AKT1/PI3K pathway. PSAE reduced ATM and GADD45A expression relative to the Model group, suggesting its potential anti-DNA damage effects through ATM inhibition and subsequent GADD45A downregulation. PSAE treatment notably decreased UVA-induced MMP1 expression while increasing the expression of key collagen genes COL1A1 and COL3A compared to the Model group (Kim, 2016). As shown in Figure 5B, ELISA analysis of cell supernatants yielded similar results. The high-dose PSAE group significantly increased the secretion of COL1 and COL3 in HFF cells, which, together with the upregulation of COL1A1 and COL3A1 gene expression, suggests that PSAE promotes collagen synthesis.

Western blot detection results shown in Figures 5F, G demonstrate that the PSAE treatment group, relative to the Model group, significantly decreased the protein expression levels of mTOR, IRS1/P-IRS1, PI3K/P-PI3K, and AKT/P-AKT, while significantly increasing the expression levels of FOXO/P-FOXO and Nrf2. These findings align with the impact of PSAE on gene expression.

### 3.5 Antiphotoaging effect of PSAE on UVA-exposed 3D reconstructed human full T-Skin™

**FIGURE 6:**
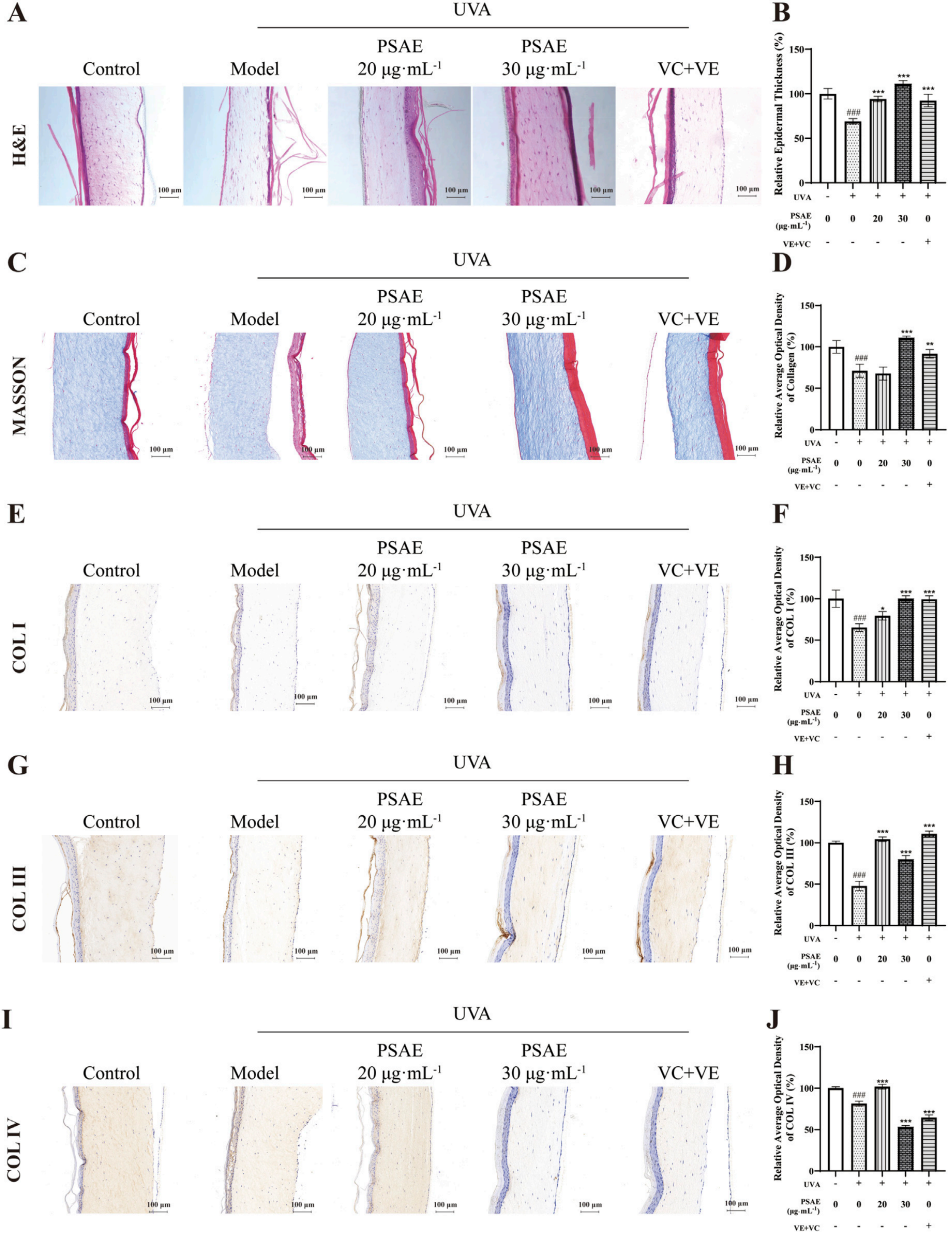
Tissue morphology study of the anti-photoaging effects of PSAE on the UVA-induced 3D full T-Skin™ photoaging model. **(A, B)** The effect of PSAE on 3D full T-Skin^™^ tissue thickness was assessed using H&E staining, with Bar = 100 μm. **(C, D)** The effect of PSAE on 3D full T-Skin™ tissue collagen content was assessed using masson staining, with Bar = 100 μm. **(E–J)** The effect of PSAE on the content of COLI **(E, F)**, COLIII **(G, H)**, and COLIV **(I, J)** in 3D full T-Skin™ tissue was assessed using IHC staining, with Bar = 100 μm.

The H&E staining results are shown in the Figures 6A, B. UVA irradiation significantly reduced the thickness of 3D skin tissue compared to the Control group, resulting in a loose epidermal structure and altered tissue morphology. The PSAE group exhibited a significant increase in 3D skin tissue thickness compared to the Model group, suggesting PSAE’s protective role against UVA-induced photoaging.

The Masson’s Trichrome staining results are shown in the Figures 6C, D. The content of collagen fibers in the PSAE group significantly increased compared to the Model group. Additionally, the immunohistochemical staining results shown in the Figures 6E–J revealed that the content of COL I COL III and COL IV were markedly elevated in PSAE groups relative to the model group. These findings suggest that PSAE has a potent ability to promote collagen synthesis and exhibits anti-photoaging effects.

**FIGURE 7:**
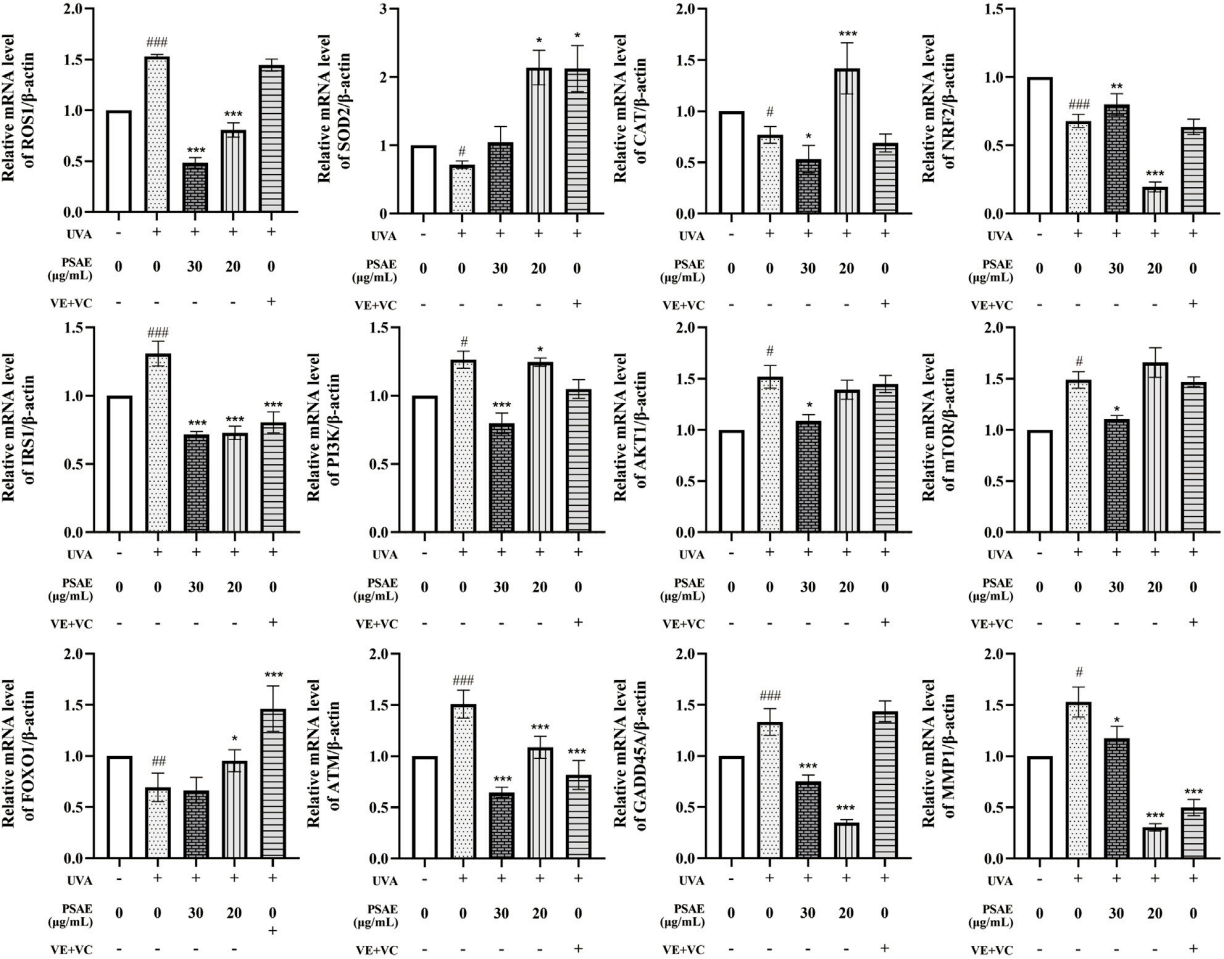
The mRNA levels study of the anti-photoaging effects of PSAE on the UVA-induced 3D full T-Skin™ photoaging model The effect of PSAE on the mRNA levels of ROS1, SOD2, CAT, Nrf2, IRS1, PI3K, AKT1, mTOR, FOXO, ATM, GADD45A, and MMP1 was measured using RT-qPCR, n = 4. Results were normalized to the blank control group and are presented as mean ± SEM. Compared to the blank control group, #*p* < 0.05, ##*p* < 0.01, ###*p* < 0.001; compared to the UVA irradiation model group, *p < 0.05, **p < 0.01, ***p < 0.001.

RT-qPCR analysis results are shown in Figure 7. In the UVA-irradiated 3D skin photoaging model, PSAE demonstrated antioxidant properties by significantly decreasing ROS1 expression and increasing the expression of antioxidants SOD2, CAT, and the oxidative stress-related factor Nrf2 compared to the Model group. PSAE modulates the IRS1/AKT1/PI3K pathway by downregulating IRS1, AKT1, PI3K, and mTOR, while upregulating FOXO, indicating that it promotes FOXO expression and inhibits mTOR expression. PSAE reduced ATM and GADD45A expression relative to the Model group, suggesting its potential anti-DNA damage effects through ATM inhibition and subsequent GADD45A downregulation. PSAE markedly reduced MMP1 expression relative to the Model group.

**FIGURE 8:**
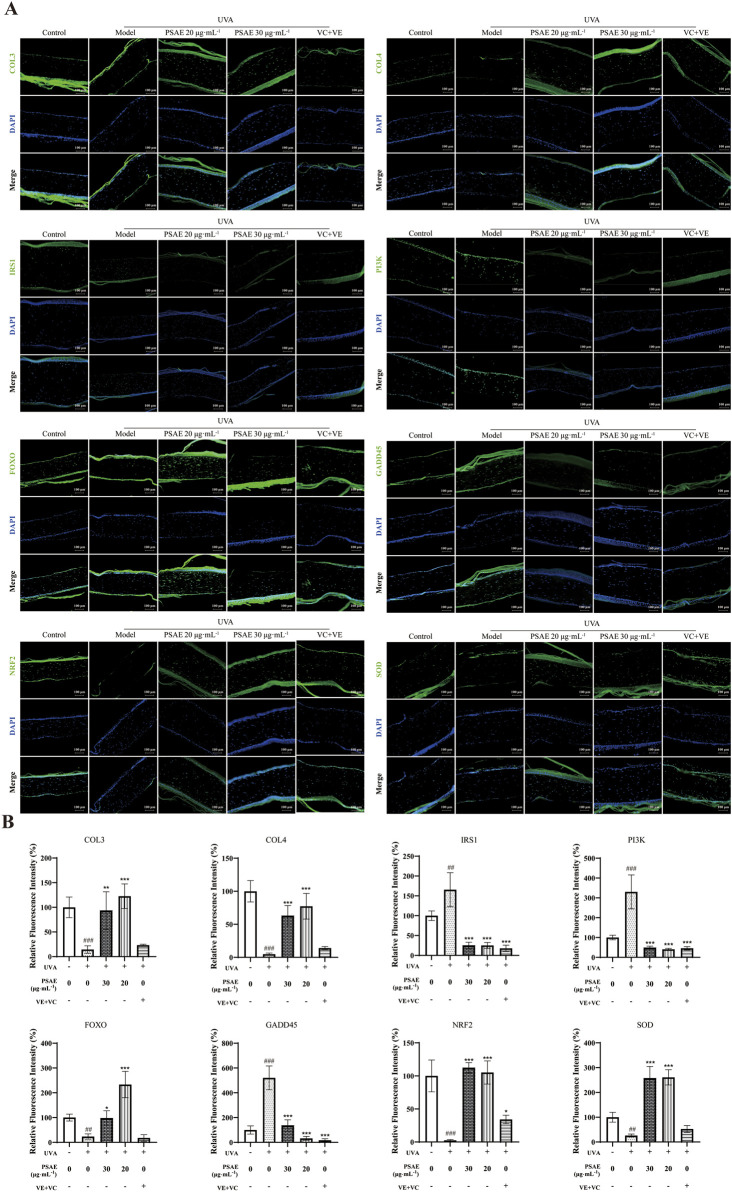
**(A)** IF detection of the effects of PSAE on the relative fluorescence intensity of COL3, COL4, IRS1, PI3K, FOXO, GADD45, Nrf2, and SOD in the UVA-irradiated 3D full T-Skin™ photoaging model. n = 3, Bar = 100 μm. **(B)** Results were normalized to the blank control group and are presented as mean ± SEM. Compared to the blank control group, #*p* < 0.05, ##*p* < 0.01, ###*p* < 0.001; compared to the UVA irradiation model group, *p < 0.05, **p < 0.01, ***p < 0.001.

Figures 8A, B illustrates the immunofluorescence detection outcomes. The PSAE group showed a marked increase in the fluorescence intensity of COL3, COL4, FOXO, NRF2, and SOD, while GADD45, IRS1, and PI3K fluorescence intensity significantly decreased compared to the Model group. These results, along with the effects of PSAE on gene expression and protein levels in the HFF cell photoaging damage model, suggest that PSAE promotes collagen synthesis, exhibits antioxidant properties, and protects against DNA damage, providing comprehensive anti-photoaging effects through the IRS1/PI3K pathway.

### 3.6 Human efficacy evaluation

Skin roughness Ra and Rq were measured using the Antera 3D system, with lower values indicating smoother skin. Trans-epidermal water loss (TEWL) was assessed using a VapoMeter, with lower values indicating better skin barrier function. Skin elasticity R2 and F4 were measured using a Cutometer. R2 represents the total amount of elastic and plastic deformation during the rebound phase relative to the stretch phase, with higher values indicating better elasticity. F4 measures skin firmness, with lower values indicating greater resistance to suction. Epidermal moisture content was measured using a Corneometer CM825, with higher values indicating increased moisture content in the stratum corneum.

**FIGURE 9:**
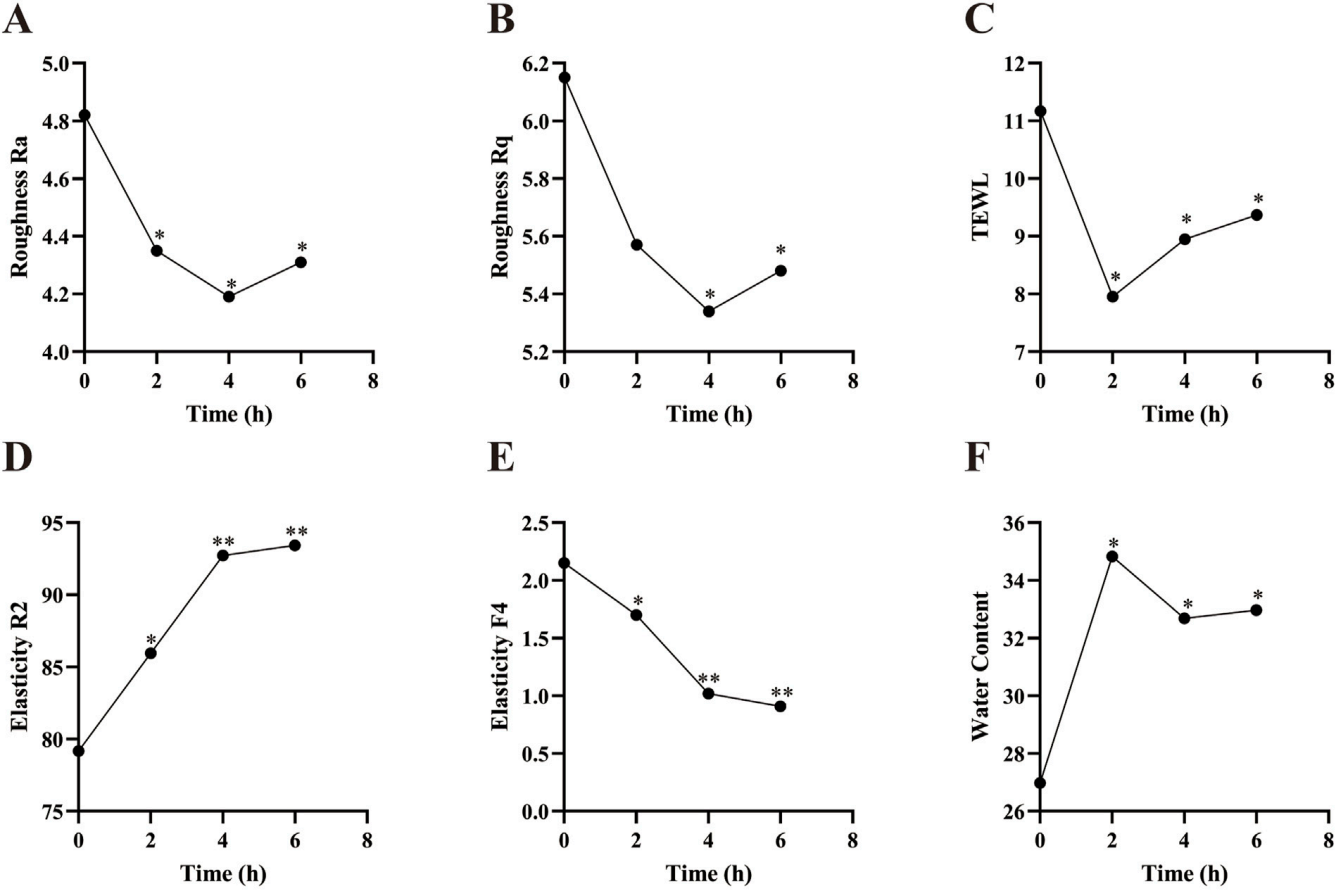
Efficacy of PSAE on human skin. **(A)** Roughness Ra **(B)** Roughness Rq **(C)** TEWL **(D)** Elasticity R2 **(E)** Elasticity F4 **(F)** Epidermal moisture content. Compared to the baseline value at T0, *p < 0.05, **p < 0.01.

As shown in Figure 9, Skin roughness Ra showed a significant reduction at 2 h, 4 h, and 6 h post-PSAE application compared to the baseline (T0). Skin roughness Rq significantly decreased at 4 and 6 h post-PSAE application, demonstrating PSAE’s effectiveness in reducing skin roughness. Following PSAE application, trans-epidermal water loss (TEWL) significantly decreased at 2, 4, and 6 h compared to the baseline (T0). Additionally, the epidermal moisture content significantly increased at 2 h, 4 h, and 6 h after PSAE application, indicating that PSAE has moisturizing effects.

Compared to the baseline value (T0), the skin elasticity parameter R2 significantly increased at 2 h, 4 h, and 6 h after PSAE application. The skin elasticity parameter F4 showed a significant decrease at 2 h, 4 h, and 6 h post-PSAE application, suggesting enhanced skin elasticity due to PSAE.

The skin roughness parameters Ra and Rq represent the average roughness and root mean square roughness, respectively, and comprehensively characterize the improvement in skin roughness due to PSAE. TEWL is an important indicator of skin barrier integrity. Research consistently indicates that elevated TEWL values are associated with compromised skin barrier function. Combined with PSAE’s ability to increase epidermal moisture content, this indicates that PSAE has moisturizing properties and can repair skin barrier damage. The skin elasticity parameter R2 is considered to be the closest approximation of the true state of skin elasticity, with higher values indicating better elasticity. F4 represents skin firmness, with lower values indicating better elasticity. PSAE significantly increased R2 and decreased F4, suggesting that PSAE improves skin elasticity (Czajka et al., 2018; Togawa et al., 2023; Kapoor et al., 2023; Wang et al., 2024).

## 4 Discussion

**FIGURE 10:**
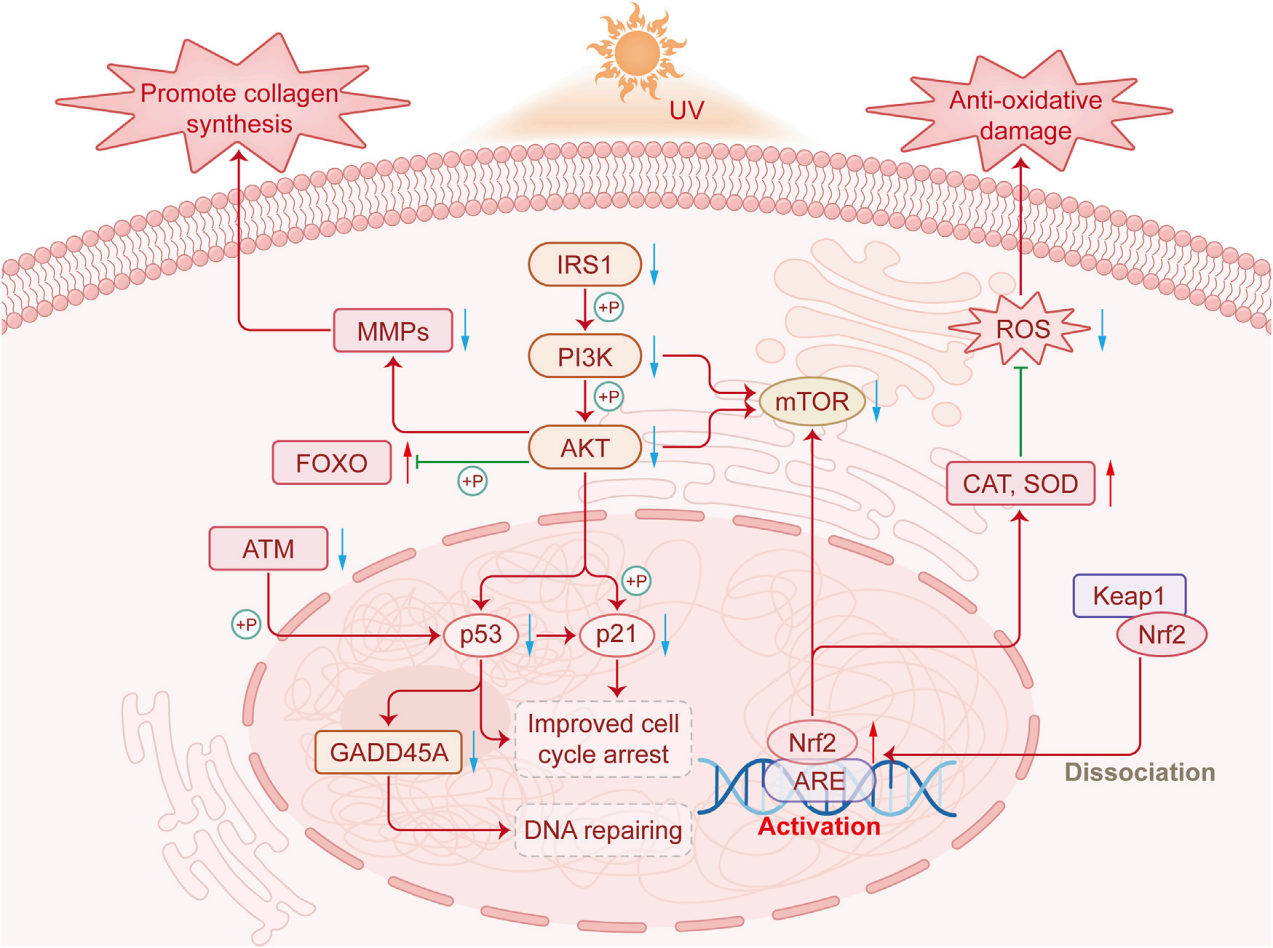
The mechanism by which PSAE improves photodamage by regulating the IRS1/PI3K/FOXO signaling pathway.

This study investigates the anti-photoaging mechanism of PSAE through various models, contributing to foundational research on skin photoaging. This study systematically explores how PSAE mitigates UV-induced photoaging damage via the IRS/PI3K/FOXO signaling pathway, as depicted in Figure 10. Reactive oxygen species (ROS) mainly consist of hydroxyl radicals, superoxide anions, and hydrogen peroxide. UV irradiation generates ROS in the skin, causing oxidative stress damage, a primary clinical sign of photoaging. This result suggests that PSAE has antioxidant properties, capable of eliminating various ROS to prevent oxidative stress induced by ROS, thereby counteracting skin photoaging (Takatsuka et al., 2022). PSAE can inhibit the activity of hyaluronidase and collagenase. Hyaluronidase and collagenase degrade hyaluronic acid and collagen, respectively. External factors like UV exposure and aging reduce hyaluronic acid and collagen levels, deteriorating skin structure and function. PSAE’s ability to inhibit hyaluronidase and collagenase indicates that it can promote the biosynthesis of collagen and hyaluronic acid, effectively maintaining skin structure and function (Yoshihara et al., 2024). UVA and UVB, as the main exogenous factors of photoaging, can directly affect cellular differentiation, growth, and aging, and lead to the degeneration of tissue function (Gromkowska-Kępka et al., 2021). This study found that UVA-induced photodamage in HFF cells is more pronounced, leading to the use of β-gal to detect cellular photoaging changes. The study found that PSAE at concentrations of 20–40 μg mL^−1^ effectively reduced ROS levels in cellular photoaging model. This characteristic suggests that PSAE can improve the related skin aging process by reducing ROS levels induced by UV radiation (Guo et al., 2022). PSAE may inhibit the phosphorylation of IRS1, thereby suppressing the phosphorylation of the PI3K/AKT pathway, increasing FOXO phosphorylation levels, and significantly reducing the protein levels of mTOR and Nrf2.

FOXO is a conserved transcriptional regulator and a downstream component of the IRS1/PI3K/AKT signaling pathway. The IRS1/PI3K/FOXO pathway regulates various physiological processes, including proliferation, apoptosis, and insulin resistance. The IRS1/PI3K/FOXO signaling pathway is activated by diverse stimuli, including ROS. Stitt et al. found that the PI3K/Akt pathway is crucial in repressing FOXO transcription factors by downregulating ubiquitin ligase expression (Gong et al., 2019). The mTOR signaling pathway regulates both energy metabolism and cellular inflammation. In research, IRS1 promotes PI3K and AKT phosphorylation to positively regulate mTOR signaling (Zheng et al., 2024). Prior studies indicate that Nrf2 is beneficial in safeguarding the skin against UVB-induced inflammation, oxidative damage, cellular dysfunction, and sunburn. Nrf2 deficiency worsens UVB-induced skin damage, such as inflammation, DNA damage, and extracellular matrix deterioration, whereas Nrf2 activation protects against UVB-induced skin cancer (Knatko et al., 2015). The Nrf2 signaling pathway regulates various oxidative stress damages in cells. Since PSAE effectively inhibits ROS production in UV-radiated HaCaT cells, it is hypothesized that PSAE may suppress UV-induced oxidative damage in skin cells via the Nrf2 signaling pathway (Gao et al., 2020). DNA damage, a hallmark of photoaging, triggers the DNA damage signaling pathway, leading to crucial cellular responses. The ATM-mediated signaling pathway is a key component in triggering cellular DNA damage responses. ATM can modulate the pro-apoptotic cell cycle regulator GADD45A by phosphorylating p53 (Jang et al., 2010). Changes in collagen and elastin in the extracellular matrix (ECM) are primary causes of clinical manifestations of skin aging, such as wrinkles, sagging, and laxity (Fisher et al., 2014). PSAE is capable of inhibiting the UVA irradiation-induced reduction in collagen content, including types I, III, and IV. Additionally, the atrophy of collagen and elastin fibers in skin aging is mainly due to increased expression of their degrading enzymes, with MMP-1 being a major MMP in UV-exposed skin. MMPs are responsible for the degradation of fibrillar collagen and elastin, which are crucial for dermal strength and elasticity. UV-induced ROS activate signaling pathways that lead to MMP overexpression and ECM degradation in connective tissues. MMP-1 inhibitors are recognized as potential agents for preventing photoaging and wrinkle formation. The results of this study show that PSAE treatment significantly downregulated the expression of UVB-induced MMP1 (Kim, 2016).

## 5 Conclusion

Based on previous research findings, the *P*. *suffruticosa* Andr. root has been widely used as a classic antioxidant and anti-aging agent in pharmaceutical and cosmetic formulations. This study innovatively developed the application of PSAE under a unique process for combating photoaging. The study explains how PSAE alleviates UV-induced photoaging damage by modulating the IRS/PI3K/FOXO signaling pathway, offering robust theoretical support for its anti-aging applications. The application of it in cosmetic ingredients has been expanded.

## Data Availability

The data supporting the conclusions of this article will be made available by the authors, without undue reservation.

## Abbreviations

ABTS, 2,2′-Azino-bis (3-ethylbenzothiazoline-6-sulfonic acid); AD, Atopic dermatitis; ANOVA, Analysis of variance; AP-1, Activator protein-1; CCK-8, Cell-countingkit-8; DPPH, 2, 2′-diphenyl-1-picrylhydrazyl; ECM, Extracellular matrix; ELISA, Enzyme-linked immunosorbent assay; H&E, Hematoxylin and eosin; HO-1, Heme oxygenase-1; HFF, Human foreskin fibroblasts; HPLC, High-performance liquid chromatography; HYP, Hydroxyproline; IF, Immunofluorescence; MDA, Malondialdehyde; MMP-1, Matrix metalloproteinase 9; NGDC, National Genomics Data Center; PSAE, *Paeonia suffruticosa* Andrews root extract; ROS, Reactive oxygen species; RT-qPCR, Real-time reverse transcription-quantitative polymerase chain reaction; SA-β-gal, Senescence-associated β-galactosidase; SOD, Superoxide dismutase; TGF-β, Transforming growth factor-β; TEWL, Transepidermal water loss; UV, Ultraviolet; UVA, Ultraviolet radiation a; UVB, Ultraviolet radiation b; VC, Vitamin C; WB, Western blotting.
