# Supplementary material for: Paeonia suffruticosa Andrews root extract ameliorates photoaging via regulating IRS1/PI3K/FOXO pathway
Source: Front Pharmacol. 2025 Mar 6;16:1520392. doi: 10.3389/fphar.2025.1520392 (PMC11923548; doi:10.3389/fphar.2025.1520392)
Supplement: Supplementary file 1 [file DataSheet1.docx]

**Supplementary Table 1. Human Sequences of the Primers**

| **Target gene** | **Sequence** |
| --- | --- |
| ***β-actin*** | Forward: 5′ CTCGCCTTTGCCGATCC 3′ |
|  | Reverse: 5′ ATCCTTCTGACCCATGCCC 3′ |
| ***SOD2*** | Forward: 5′ GGAAGCCATCAAACGTGACTT 3′ |
|  | Reverse: 5′ CCCGTTCCTTATTGAAACCAAGC 3′ |
| ***GADD45A*** | Forward: 5′ GAGAGCAGAAGACCGAAAGGA 3′ |
|  | Reverse: 5′ CACAACACCACGTTATCGGG 3′ |
| ***CAT*** | Forward: 5′ TGGAGCTGGTAACCCAGTAGG 3′ |
|  | Reverse: 5′ CCTTTGCCTTGGAGTATTTGGTA 3′ |
| ***ATM*** | Forward: 5′ TTGATCTTGTGCCTTGGCTAC 3′ |
|  | Reverse: 5′ TATGGTGTACGTTCCCCATGT 3′ |
| ***ROS1*** | Forward: 5′ATGCAGACCTACCAACTGCTC 3′ |
|  | Reverse: 5′ GGGCTTGACCACATAGGACG 3′ |
| ***IRS1*** | Forward: 5′ ACAAACGCTTCTTCGTACTGC 3′ |
|  | Reverse: 5′ AGTCAGCCCGCTTGTTGATG 3′ |
| ***PIK3CB*** | Forward: 5′ TATTTGGACTTTGCGACAAGACT 3′ |
|  | Reverse: 5′ TCGAACGTACTGGTCTGGATAG 3′ |
| ***AKT1*** | Forward: 5′ GTCATCGAACGCACCTTCCAT 3′ |
|  | Reverse: 5′ AGCTTCAGGTACTCAAACTCGT 3′ |
| ***FOXO1*** | Forward: 5′ TCGTCATAATCTGTCCCTACACA 3′ |
|  | Reverse: 5′ CGGCTTCGGCTCTTAGCAAA 3′ |


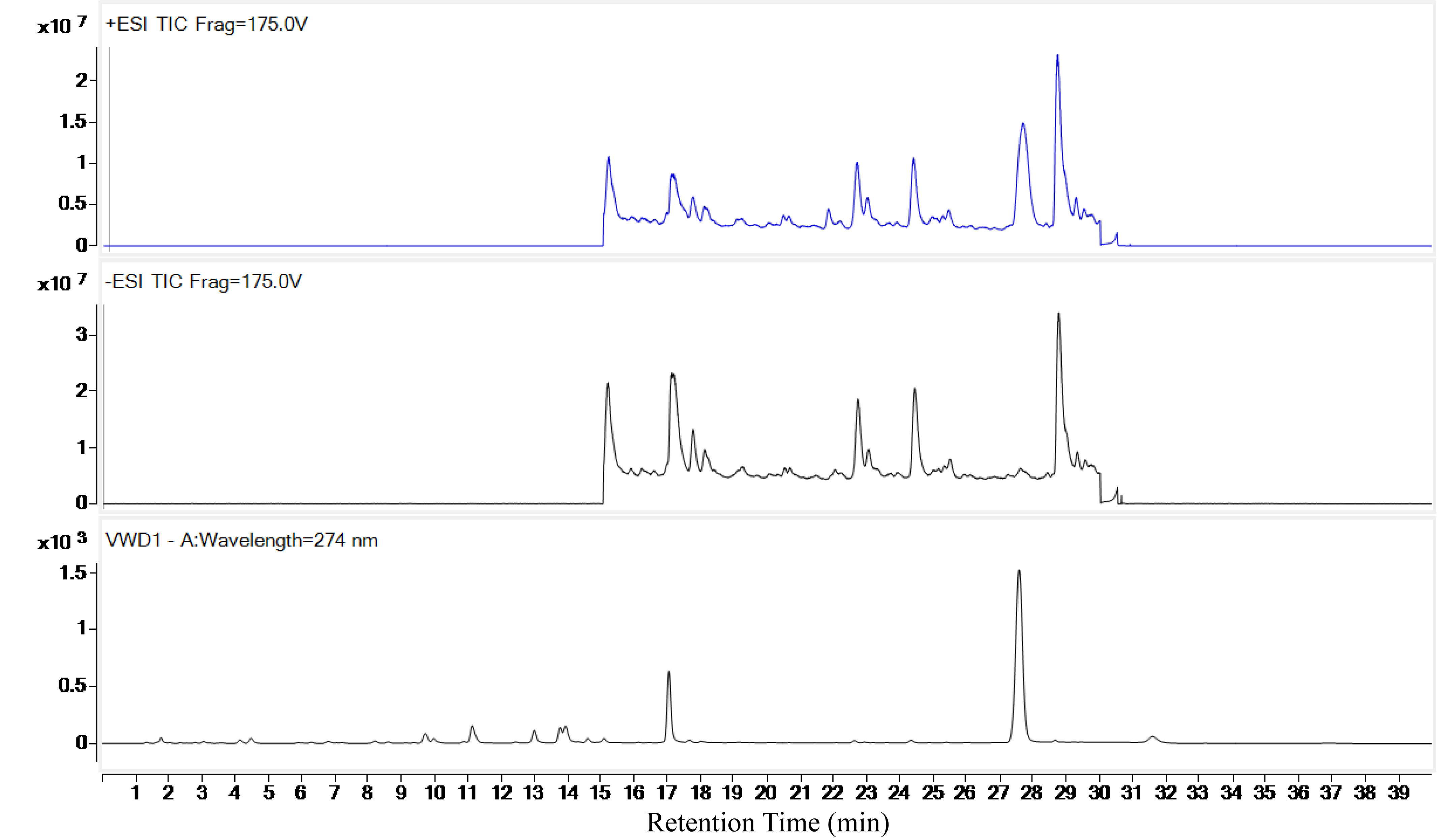


**Supplemental Figure S1-1. Total ion chromatogram (TIC) in both positive and negative ion modes and HPLC chromatogram of PSAE.**

**A**

**B**


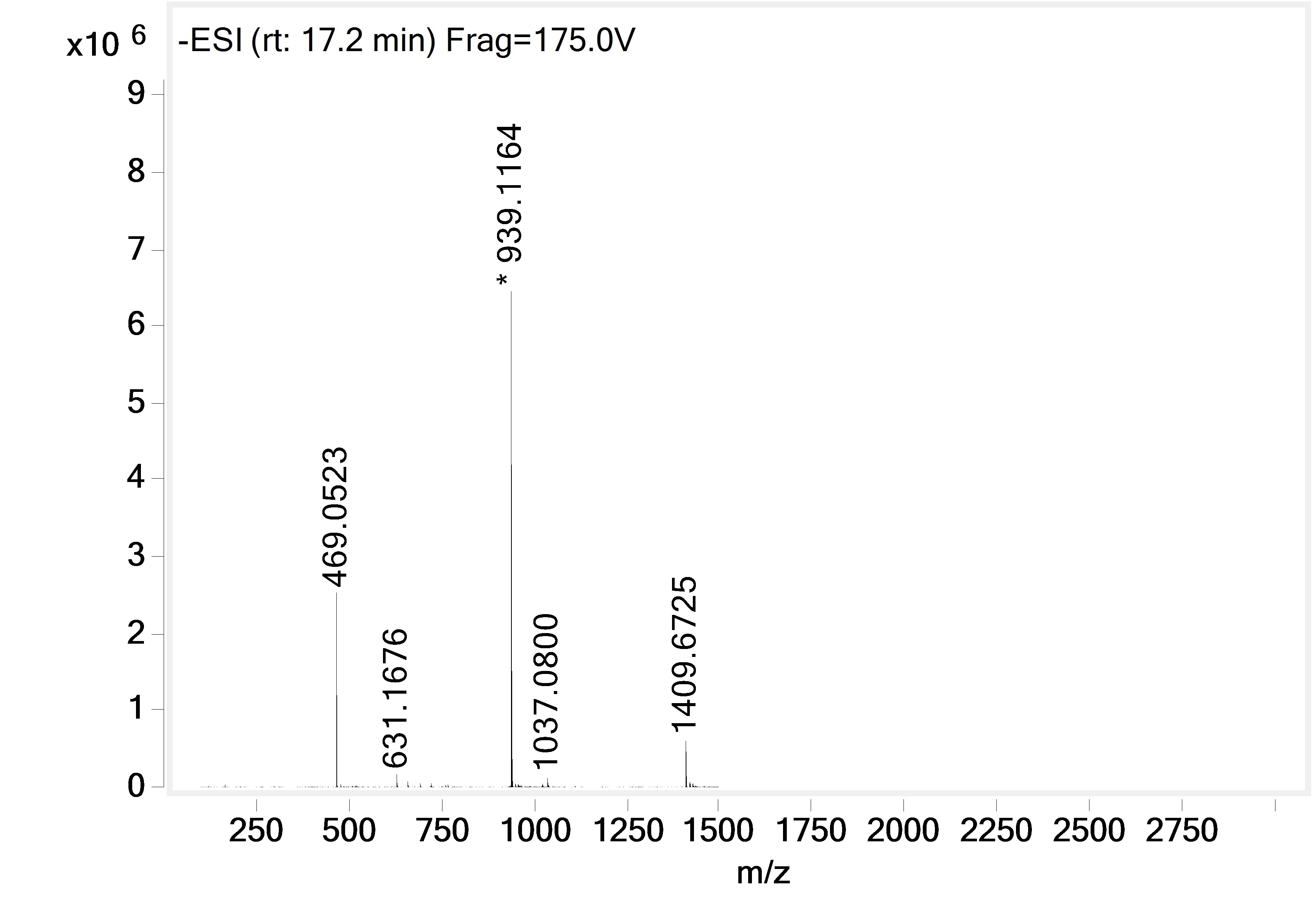

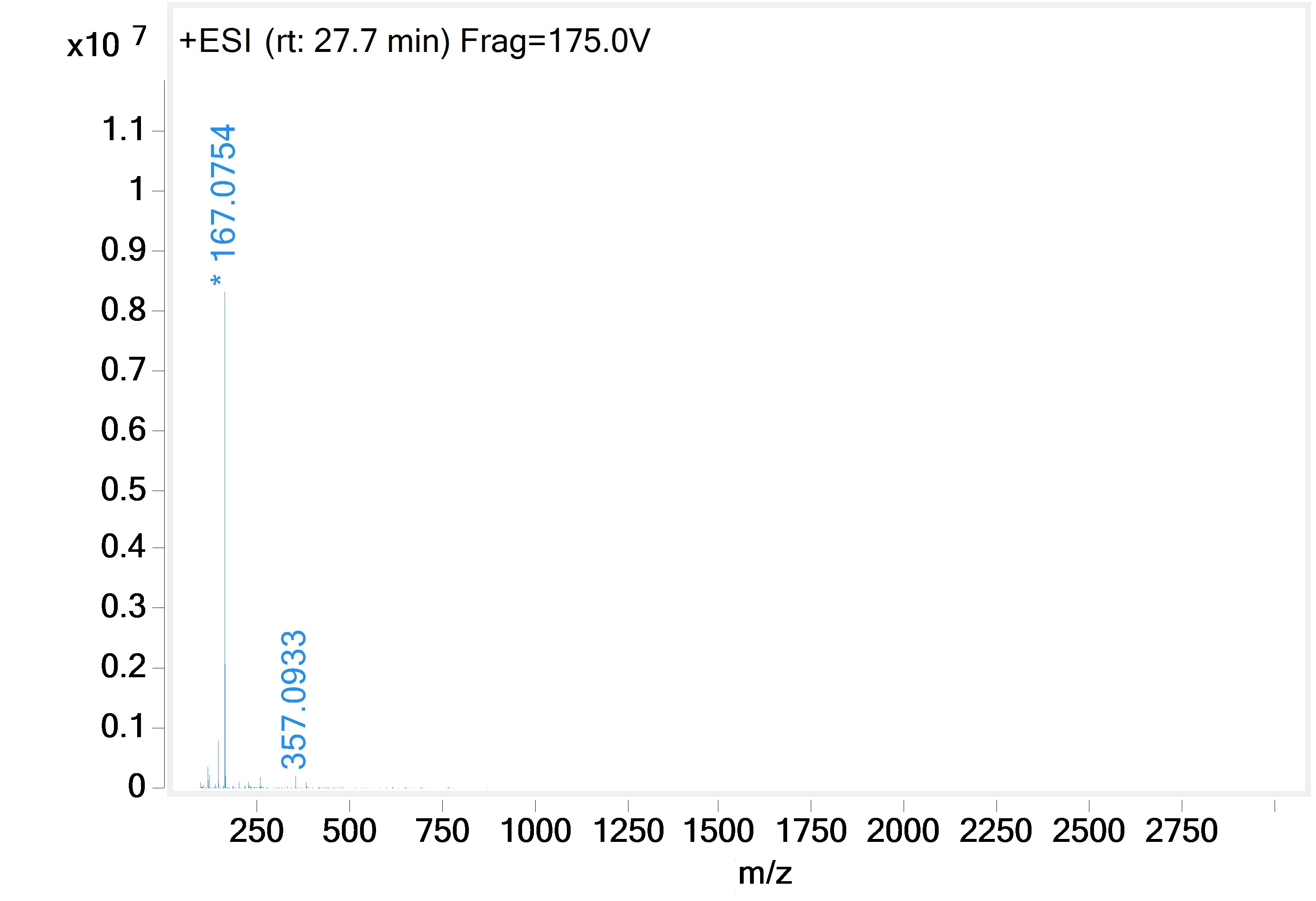


**C**


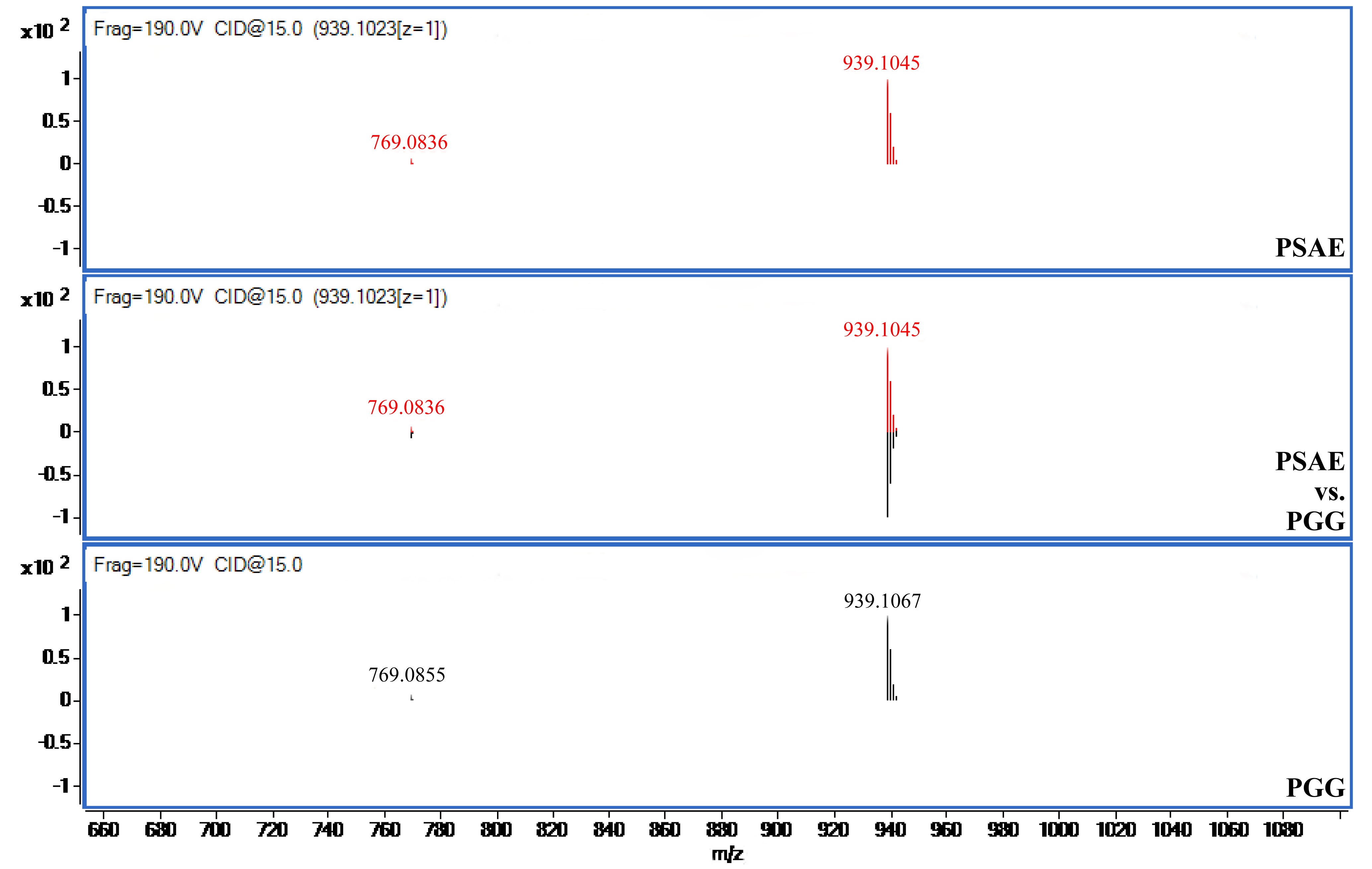


**D**


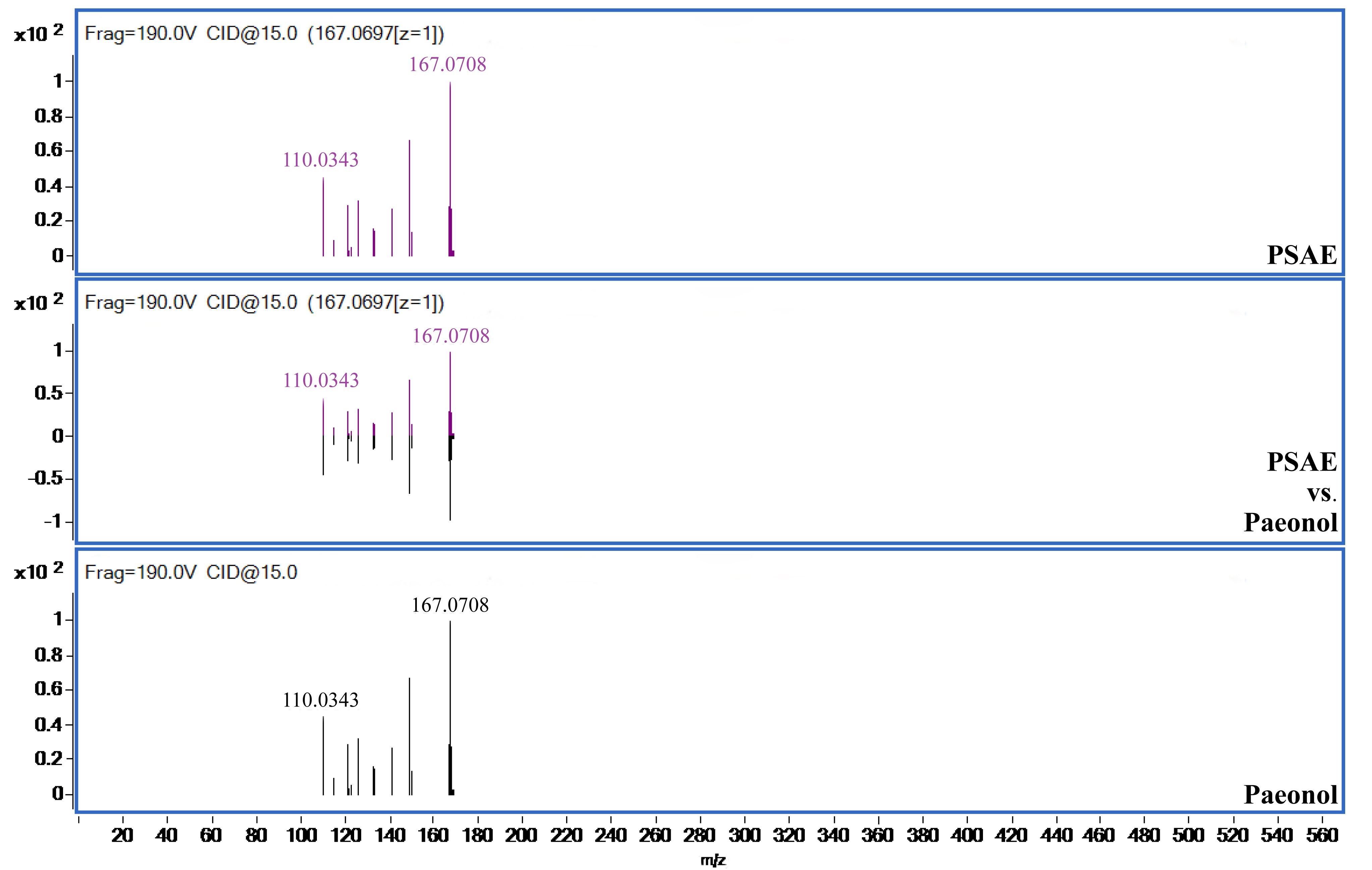


**Supplemental Figure S1-2. Mass spectrometry diagrams of the HPLC chromatographic peaks corResponseing to A: PSAE-PGG (rt=17.2min; [M-H] ^-^ = 939.1164) B: PSAE-paeonol (rt=27.7min; [M+H]^+^ = 167.0754) C:** **Comparison characteristic secondary fragments in mass spectrometry chart of PGG standard sample and PSAE-PGG D: Comparison characteristic secondary fragments in mass spectrometry chart of paeonol standard sample and PSAE-paeonol**

| Protein | Dark Field | Bright Field | Exposure |
| --- | --- | --- | --- |
| MTOR（Repeat 1） |  | 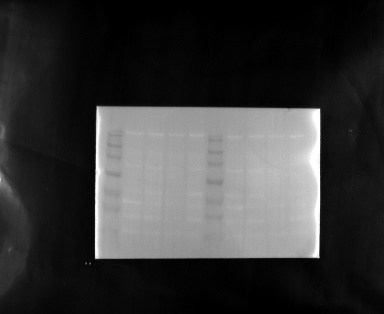 |  |
| MTOR（Repeat 2） |  | 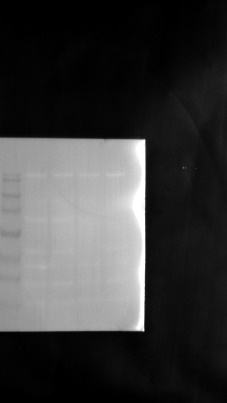 |  |
| MTOR（Repeat 3） |  | 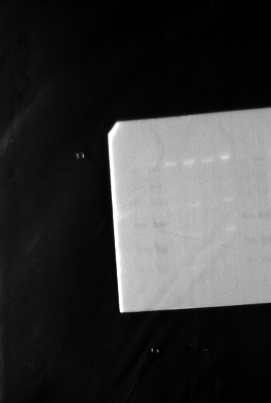 |  |
| P-IRS1（Repeat 1） |  | 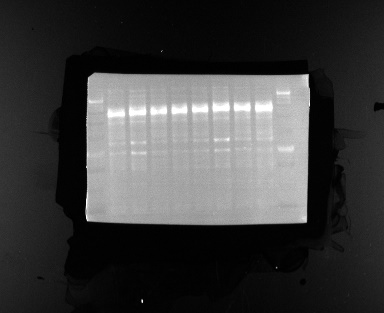 |  |
| P-IRS1（Repeat 2） |  | 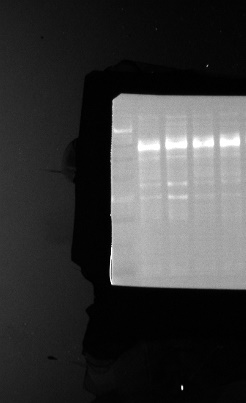 |  |
| P-IRS1（Repeat 3） |  | 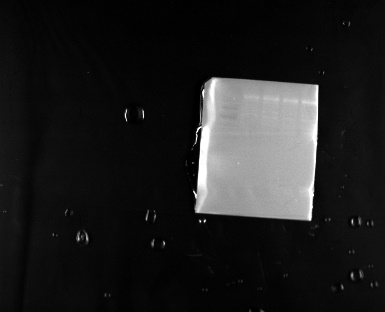 |  |
| IRS1  （Repeat 1） |  | 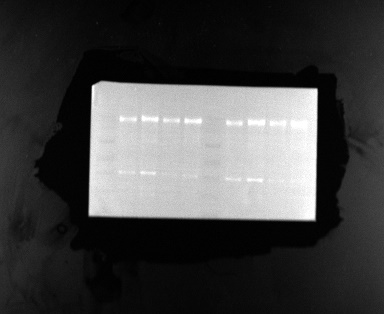 |  |
| IRS1  （Repeat 2） |  | 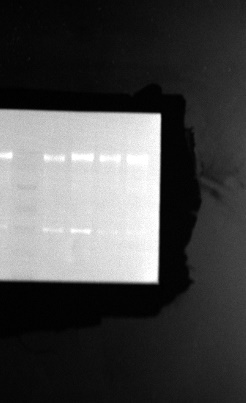 |  |
| IRS1  （Repeat 3） |  | 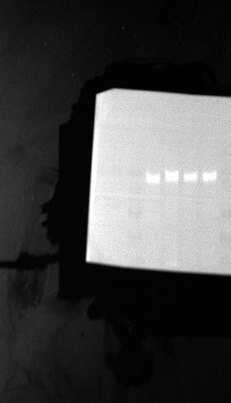 |  |
| P-PI3K（Repeat 1） |  | 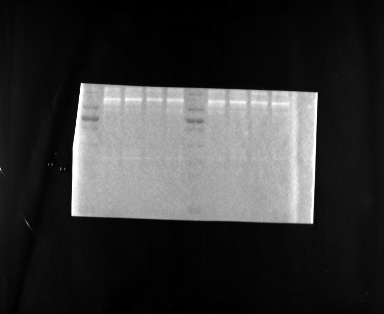 |  |
| P-PI3K（Repeat 2） |  | 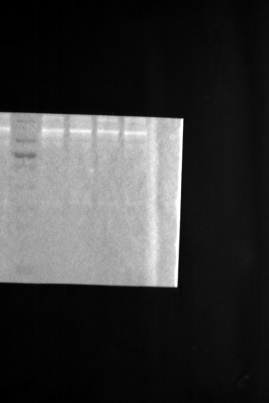 |  |
| P-PI3K（Repeat 3） |  | 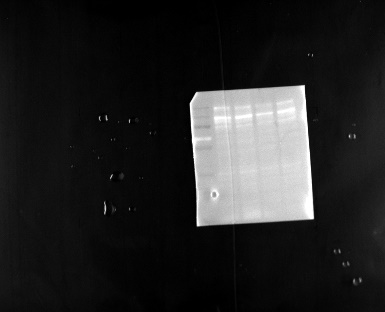 |  |
| PI3K  （Repeat 1） |  | 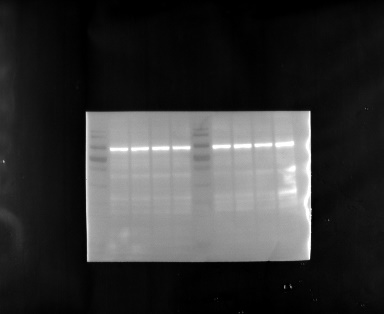 |  |
| PI3K  （Repeat 2） |  | 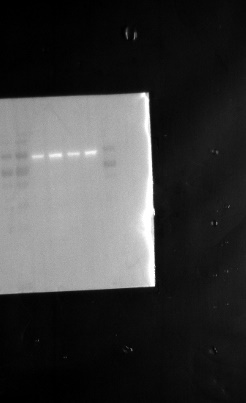 |  |
| PI3K  （Repeat 3） |  | 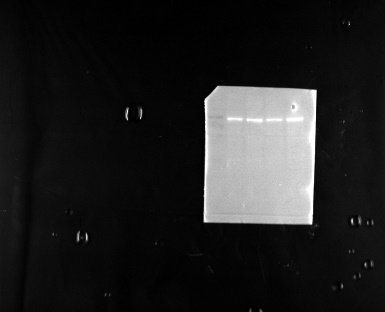 |  |
| P-FOXO（Repeat 1） |  | 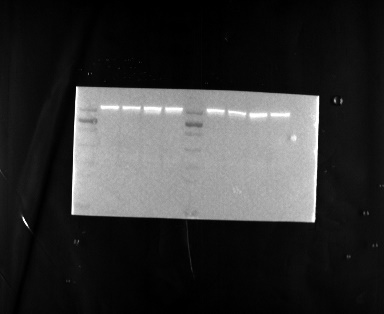 |  |
| P-FOXO（Repeat 2） |  | 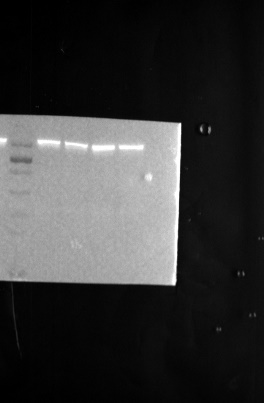 |  |
| P-FOXO（Repeat 3） |  | 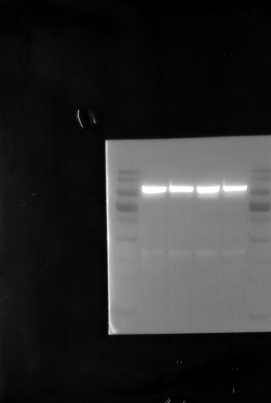 |  |
| FOXO（Repeat 1） |  | 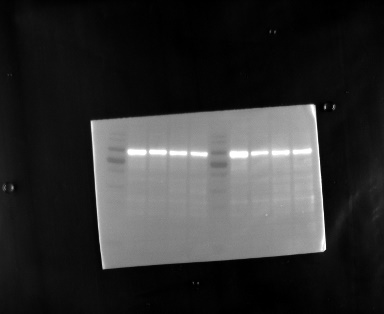 |  |
| FOXO（Repeat 2） |  | 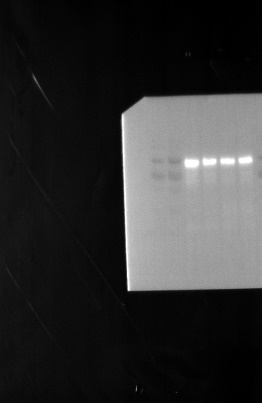 |  |
| FOXO（Repeat 3） |  | 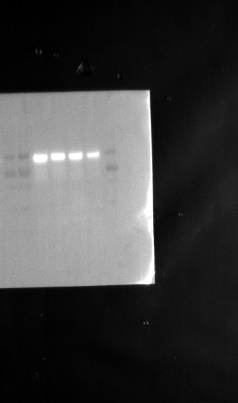 |  |
| NRF2（Repeat 1） |  | 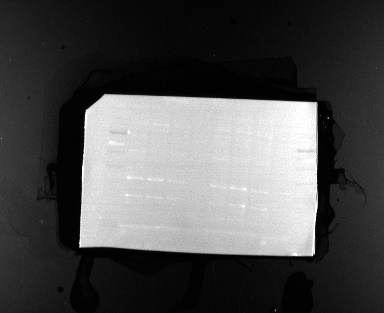 |  |
| NRF2（Repeat 2） |  | 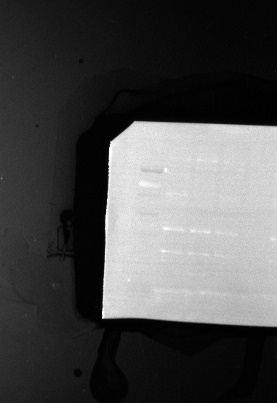 |  |
| NRF2（Repeat 3） |  | 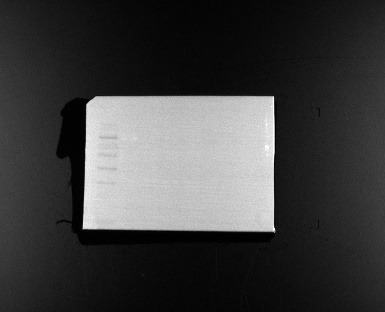 |  |
| P-AKT（Repeat 1） |  | 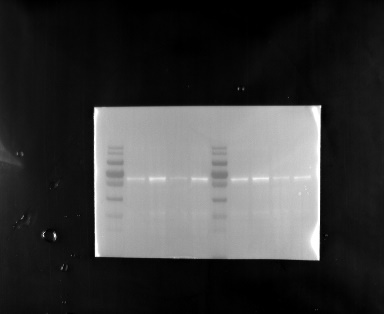 |  |
| P-AKT（Repeat 2） |  | 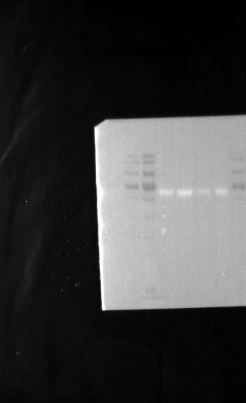 |  |
| P-AKT（Repeat 3） |  | 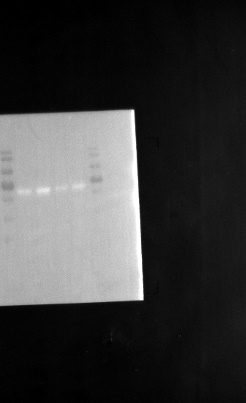 |  |
| AKT  （Repeat 1） |  | 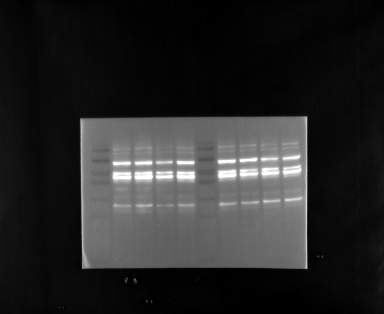 |  |
| AKT  （Repeat 2） |  | 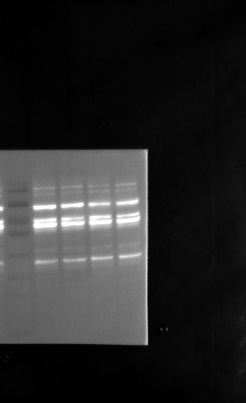 |  |
| AKT  （Repeat 3） |  | 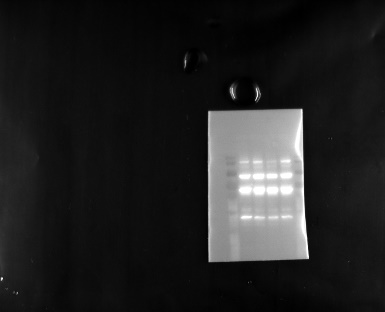 |  |
| β-actin（Repeat 1） |  | 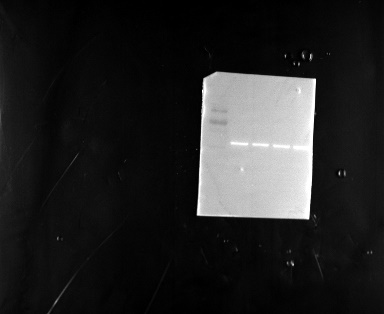 |  |
| β-actin（Repeat 2） |  | 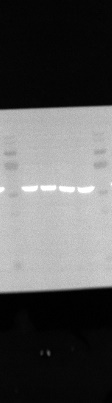 |  |
| β-actin（Repeat 3） |  |  |  |

**Supplemental Figure S2-1. Western blot analysis image of UVB-induced HaCaT cells photoaging model**

| Protein | Dark Field | Bright Field | Exposure |
| --- | --- | --- | --- |
| MTOR（Repeat 1） |  |  |  |
| MTOR（Repeat 2） |  |  |  |
| MTOR（Repeat 3） |  |  |  |
| P-IRS1（Repeat 1） |  |  |  |
| P-IRS1（Repeat 2） |  |  |  |
| P-IRS1（Repeat 3） |  |  |  |
| IRS1  （Repeat 1） |  |  |  |
| IRS1  （Repeat 2） |  |  |  |
| IRS1  （Repeat 3） |  |  |  |
| P-PI3K（Repeat 1） |  |  |  |

| P-PI3  （Repeat 2） |  |  |  |
| --- | --- | --- | --- |
| P-PI3K（Repeat 3） |  |  |  |

| PI3K  （Repeat 1） |  |  |  |
| --- | --- | --- | --- |

| PI3K  （Repeat 2） |  |  |  |
| --- | --- | --- | --- |
| PI3K  （Repeat 3） |  |  |  |

| P-FOXO（Repeat 1） |  |  |  |
| --- | --- | --- | --- |

| P-FOXO（Repeat 2） |  |  |  |
| --- | --- | --- | --- |
| P-FOXO（Repeat 3） |  |  |  |

| FOXO（Repeat 1） |  |  |  |
| --- | --- | --- | --- |

| FOXO（Repeat 2） |  |  |  |
| --- | --- | --- | --- |
| FOXO（Repeat 3） |  |  |  |

| NRF2（Repeat 1） |  |  |  |
| --- | --- | --- | --- |

| NRF2（Repeat 2） |  |  |  |
| --- | --- | --- | --- |
| NRF2（Repeat 3） |  |  |  |

| P-AKT（Repeat 1） |  |  |  |
| --- | --- | --- | --- |

| P-AKT（Repeat 2） |  |  |  |
| --- | --- | --- | --- |
| P-AKT（Repeat 3） |  |  |  |

| AKT  （Repeat 1） |  |  |  |
| --- | --- | --- | --- |

| AKT  （Repeat 2） |  |  |  |
| --- | --- | --- | --- |
| AKT  （Repeat 3） |  |  |  |

| β-actin（Repeat 1） |  |  |  |
| --- | --- | --- | --- |

| β-actin（Repeat 2） |  |  |  |
| --- | --- | --- | --- |
| β-actin（Repeat 3） |  |  |  |

**Supplemental Figure S2-2. Western blot analysis image of UVA-induced HFF cells photoaging model**
